# Effectiveness of cold-water immersion vs. massage in reducing delayed-onset muscle soreness and enhancing recovery following CrossFit^®^ Murph Workout: Randomized rial

**DOI:** 10.1371/journal.pone.0329892

**Published:** 2025-08-13

**Authors:** Gabrielly Santos Pereira, Giovana Eduarda Paulino, Thais Helena Martins Faria de Almeida, Cissa Maria Ribeiro Siqueira, Josie Resende Torres da Silva, Marcelo Lourenço da Silva, Luciano Maia Alves Ferrera

**Affiliations:** 1 Laboratory of Neuroscience, Neuromodulation and Study of Pain (LANNED), UNIFAL-MG, Alfenas, Minas Gerais, Brazil; 2 Neuromodulation and Pain Lab (NeuroPain), Egas Moniz Interdisciplinary Research Center (CiiEM), Almada, Portugal; Shahrood University of Technology, IRAN, ISLAMIC REPUBLIC OF

## Abstract

The Murph workout, one of the most challenging CrossFit® workouts, demands endurance and high intensity. This WOD (Workout of the Day) includes a 1-mile run, 100 pull-ups, 200 push-ups, 300 air squats, and another 1-mile run, typically performed while wearing a weighted vest. Due to its high physical demands, athletes commonly experience Delayed Onset Muscle Soreness (DOMS), characterized by increased sensitivity, fatigue, and reduced muscle function. To minimize these effects and ensure proper recovery, it is essential to adopt strategies that restore muscle function, reduce pain, and allow athletes to return to training without an elevated risk of injury. Thus, the objective of this study was to investigate the effects of massage therapy (MAS) or cold-water immersion (CWI) as a recovery intervention for DOMS in athletes following high-intensity physical activity during the CrossFit® Murph workout. For this purpose, thirty individuals with a minimum of six months of CrossFit® experience and familiarity with all exercises used in the study were recruited. Pain assessment questionnaires, including the Brief Pain Inventory (BPI) and the A-DOM questionnaire, along with a socioeconomic questionnaire, were administered before and after WOD. Additionally, pain assessments were conducted using algometry and thermographic imaging. After completing the WOD, participants were randomly assigned to one of two recovery interventions: MAS or CWI. The study results highlight the differential impacts of CWI and MAS on pain management and recovery dynamics following structured exercise. Our findings clearly demonstrate that CWI significantly reduces pain prevalence, both at rest and during exercise, as evidenced by the absence of pain reports from participants 48 hours after the intervention. While our study provides valuable insights into the effectiveness of CWI and MAS for post-exercise recovery, limitations such as the non-blinded study design and small sample size may influence the generalizability of the findings.

## Introduction

The Murph workout, a cornerstone of CrossFit®, is one of the most physically demanding workouts of the day (WOD) [1]. It combines a one-mile run, 100 pull-ups, 200 push-ups, 300 air squats, and another one-mile run, often performed while wearing a weighted vest. This sequence mimics the physiological demands of long-distance endurance events like marathons, involving prolonged exertion, eccentric loading, and sustained high-intensity activity [2]. These demands can lead to significant fatigue, including central nervous system strain and peripheral muscle exhaustion. Effective recovery following such exertion is essential to restore muscle function, reduce soreness, and enable athletes to resume training without an elevated risk of injury or overtraining syndrome [3,4].

Delayed-onset muscle soreness (DOMS), a common consequence of high-intensity exercise, is a multifactorial condition caused by muscle damage, inflammatory responses, and impaired muscle function. As a result, effective recovery strategies are critical. Two widely used interventions are Cold Water Immersion (CWI) and Massage Therapy (MAS) [5]. CWI has been shown to reduce muscle soreness by inducing vasoconstriction and decreasing metabolic activity, while MAS improves blood circulation, reduces muscle tension, and stimulates relaxation responses. Both interventions have demonstrated benefits for recovery, but their specific effects on DOMS following high-intensity functional exercises like the Murph workout have not been thoroughly compared in the literature [6–8].

Though research has extensively investigated CWI and MAS for general exercise recovery, most studies have focused on endurance or resistance training, not high-intensity functional workouts. The gap in comparative research on CWI and MAS in the context of CrossFit® workouts, particularly the Murph workout, underscores the need for this study [9–11]. Existing studies on CWI have shown inconsistent results, with some indicating minor improvements in power recovery, but with significant variability and methodological limitations. Similarly, while MAS is popular among athletes, there is a lack of systematic research evaluating its efficacy in functional fitness contexts [3,7,12]. Both CWI and MAS are believed to reduce muscle soreness and enhance recovery, yet their mechanisms differ significantly: MAS relies on mechanical pressure to alleviate muscle stiffness and stimulate relaxation [13], while CWI uses cold-induced analgesia to reduce muscle inflammation and stiffness [10,14].

Thus, the objective of this study is to compare the effects of CWI and MAS on recovery from DOMS in athletes following the CrossFit® Murph workout. We hypothesize that CWI will significantly reduce subjective muscle soreness, pressure pain threshold (PPT), and pain interference over the 48-hour recovery period, compared to MAS. Specifically, we expect that CWI will lead to greater improvements in muscle soreness and pain reduction immediately post-intervention and at 24 and 48 hours following the workout.

## Methods

### Study design and participants

This was a randomized controlled trial conducted to assess the effects of CWI and MAS on recovery from DOMS in athletes following the CrossFit® Murph workout. Thirty participants were recruited between November 8 and November 15, 2024. All participants were free from injury and illness, were not using performance-enhancing drugs, and had at least 6 months of experience with CrossFit® and were familiar with all exercises used in the study. Participants were required to train at least five times per week, with each session consisting of 10 minutes of warm-up, 40 minutes of strength and power training, and 20 minutes of metabolic conditioning [15].

Participants were advised to refrain from consuming alcohol for 24 hours before any tests, to avoid exercise 48 hours before the workout, and to maintain their normal diet and hydration levels throughout the study. No injuries were reported prior to or during the study. All participants provided written informed consent, and the study was approved by the University Research Ethics Committee for Human Use (7.088.206; September 19, 2024). The study adhered to the Helsinki Declaration on the use of human participants for research and was registered in the Brazilian Registry of Clinical Trials (ReBEC) under the number RBR-7x8bkv2.

### Randomization and blinding

Participants were randomly assigned to one of two recovery interventions: Massage Therapy (MAS) or Cold Water Immersion (CWI). Randomization was performed using block randomization to ensure balanced group allocation. Outcome assessors were blinded to group assignments to reduce bias during the measurement phase. However, participants were aware of their assigned intervention, which may introduce a potential source of bias.

### Murph protocol method

The Murph WOD (Workout of the Day) is a well-known and demanding CrossFit® benchmark workout, typically performed for time. It consists of a series of high-intensity exercises, including a 1 mile run, 100 pull-ups, 200 push-ups, 300 air squats, and a final 1 mile run. The workout is designed to be completed as quickly as possible, with each component performed sequentially. Participants were allowed to partition the workout (e.g., 20 rounds of 5 pull-ups, 10 push-ups, and 15 squats) if they preferred, but the sequence of exercises had to be followed. The workout was performed individually for time.

### Recovery interventions

After completing the Murph workout, participants were assigned to one of the following recovery interventions:

Massage Therapy (MAS): A licensed therapist administered 20 minutes of Swedish massage targeting the upper body (arms and shoulders) and lower body (legs and back). The therapist applied moderate pressure to reduce muscle tension and improve circulation.

Cold Water Immersion (CWI): Participants immersed themselves in ice-cold water at 8°C for 20 minutes. A water temperature monitor ensured that the immersion temperature remained consistent throughout the session.

### Outcome measures

The primary outcome measures were subjective soreness (assessed using the Brief Pain Inventory (BPI) and Q-ADOM), pressure pain threshold (PPT), and muscle soreness (assessed using the Visual Analog Scale (VAS)). The secondary outcome included: Muscle inflammation (assessed using thermographic imaging of the anterior arm and dorsal leg regions). These outcomes were assessed at four timepoints: Pre: baseline, before the workout; Postrec: immediately after the intervention; Post24: 24 hours after the workout; Post48: 48 hours after the workout.

### Brief pain inventory (BPI)

BPI is a questionnaire designed to capture both pain intensity and the amount of interference that pain has on functioning [16]. BPI has been extensively used in both cancer- and non-cancer-related pain. Pain intensity is measured with four items (worst, least, on average, and currently). Interference is measured with seven items, including general activity, mood, walking, work (including paid and household work), relations with others, sleep, and enjoyment of life. The patient answers the items on a scale of 0–10, the highest number indicating the worst imaginable pain for intensity items and complete interference for interference items.

### Musculoskeletal pain assessment questionnaire (Q-ADOM)

The questionnaire consists of a series of questions structured on a Likert scale that ranges from 0 to 10. A score of “0” indicates that the practitioner feels no pain at all, while a score of “10” indicates extreme pain [17]. Scores from 0 to 3 are associated with a perception of mild pain, where no significant impact on performance is expected. Scores from 4 to 6 suggest moderate pain, which may interfere with daily activities and exercise practice, while scores from 7 to 10 indicate intense pain, likely resulting in a reduction in physical performance and adherence to the exercise program.

### Dynamic pressure algometry

Pressure pain threshold (PPT) was assessed using a portable pressure algometer with a 1 cm^2^ rubber tip, featuring a digital communication interface and applied perpendicularly to the skin at a rate of 0–50 kgf/s (PHYSIOCODE, Belo Horizonte, MG, Brazil). PPT was defined as the minimum pressure evoking the first painful sensation. PPT was measured bilaterally in the mid-belly *biceps brachii* and *gastrocnemius* muscles three times with a 10-s interval for each point, and the average value was used for statistical analysis.

### Visual analog scale (VAS)

The VAS for pain consists of five 10-cm lines, the left end labeled ‘No pain’ (0 cm) and the right end ‘Very severe pain’ (10 cm). Patients were asked to draw a vertical mark on the top line for their current pain, on the second line for their average pain during the last week, on the third line for their worst pain in the last week, on the fourth line for their lowest pain level in the last week, and on the fifth line for their average pain during the last week [18].

### Thermal images processing method

The infrared images were collected using a T430sc® infrared camera (FLIR, USA), with an image resolution of 320 x 240 pixels, uncooled microbolometer, which has sensors that allow measuring the temperatures ranging from – 20°C to +120°C, with thermal sensitivity of 0.05°C and accuracy of ± 2ºC of absolute temperature. The images were performed in a controlled room with temperature set at 20 ± 2°C, relative humidity less than 60% and skin emissivity has been set at 0.98. A digital thermo-hygrometer (Minipa® model MT241) for room temperature monitoring was used. Each region of interest (ROIs) was analyzed in terms of mean temperature. The regions used were: Anterior Arm Right (AAR); Anterior Arm Left (AAL); Dorsal Leg Right (DLR) and Dorsal Leg Left (DLL). Symmetry was analyzed between ipsilateral and its contralateral mean ROI temperature. Thermographic imaging was also used to assess muscle temperature, which could indicate inflammation or other physiological changes [19].

### Sample size calculation

A power calculation was performed using G-power software (version 3.1.9.4, Germany) to determine the appropriate sample size for statistical significance. Based on an alpha of 0.05, a power of 0.8, and an effect size of 0.4, we determined the required total sample size to be eight participants for a repeated measures analysis of variance (ANOVA). Furthermore, it has been suggested that in order to detect interaction effects between sexes, as well as main effects, the sample size needs to be four-times the size. To account for dropout rate, and to ensure adequate ability to detect interaction and main effects, we attempted to recruit approximately 15 per group for a total of 30 participants.

### Statistical analysis

Shapiro-Wilk test was used to analyze data distribution. Mean temperatures (TMe) were obtained of all ROIs studied and it was used in the analysis. Percentiles were used to characterize the sample and due to normal distribution violation, the Wilcoxon test was used to compare the skin temperature. McNemar’s test was used to show changes within each group over time; Fisher’s Exact Test was used to compare differences between groups at each time point. The level of statistical significance was set at 95%. The Statistical analyses were performed using Statistical Package for Social Sciences (SPSS, version 25.0).

## Results

The study initially recruited 30 volunteers, but only 18 successfully completed the full cycle of evaluations and were considered for the analysis (Table 1). These participants were divided into two recovery groups: Massage (MAS) and Cold Water Immersion (CWI). Participants in the MAS group (n = 8) were exclusively female, with an average age of 33.13 ± 9.02 years, weight of 69.60 ± 11.62 kg, and height of 167.88 ± 11.62 cm. In contrast, the CWI group (n = 10) included both male (n = 2) and female (n = 8) participants, with an average age of 31.88 ± 9.78 years, weight of 65.50 ± 10.43 kg, and height of 165.88 ± 8.67 cm. Overall, the study population had an average age of 32.50 ± 9.32 years, weight of 67.55 ± 10.93 kg, and height of 166.88 ± 8.83 cm. The finish time for the Murph workout demonstrated a performance edge for the CWI group (55.63 ± 4.89 minutes) over the MAS group (57.75 ± 5.32 minutes), with an overall average time of 54.19 ± 5.21 minutes across all participants. These baseline characteristics and performance metrics set the context for analyzing the effects of the respective recovery interventions on subsequent measures of physiological and thermal responses.

**Table 1:** Physical and performance characteristics of volunteers.

	Recovery groups		
	MAS (n = 8)	CWI (n = 10)	Overall (n = 18)
**Age (years)**	33.13 ± 9.02	31.88 ± 9.78	32.50 ± 9.32
**Weight (kg)**	69.60 ± 11.62	65.50 ± 10.43	67.55 ± 10.93
**Height (cm)**	167.88 ± 11.62	165.88 ± 8.67	166.88 ± 8.83
**Male**	0	8	8
**Female**	8	2	10
**Finish time (min)**	57.75 ± 5.32	55.63 ± 4.89	54.19 ± 5.21

Massage (MAS); cold water immersion (CWI).

In this study, we evaluated the effects of cold water immersion (CWI) and massage (MAS) on skin temperature and thermal asymmetry in various body regions (anterior arm right, anterior arm left, dorsal leg right, dorsal leg left) following a Murph workout. Measurements were taken at four different times: before the exercise (Pre), immediately after the recovery intervention (Postrec), and at 24 hours (Post24) and 48 hours (Post48) post-intervention.

The data from the study suggest a variable response to the different recovery interventions across measured sites (Table 2). For both CWI and MAS interventions, temperature changes exhibited distinct patterns depending on the body part and the type of recovery intervention applied.

**Table 2:** Skin temperature and thermal asymmetries measures of volunteers.

	Group	Pre			Postrec			Post24			Post48				
		Percentiles			Percentiles			Percentiles			Percentiles			Wilcoxon test	
		25º	50º	75º	25º	50º	75º	25º	50º	75º	25º	50º	75º	Z	*p value*
AAR	MAS CWI	32.40 30.79	32.87 32.13	33.41 32.81	32.48 31.15	33.16 32.13	34.00 32.97	32.53 31.20	33.09 32.11	33.87 33.08	32.41 30.90	33.03 32.03	33.75 32.87	2.0 1.0	*0.05* *0.02*
AAL	MAS CWI	30.19 30.37	31.70 31.60	32.91 32.86	30.63 29.99	31.90 31.88	33.08 33.34	30.21 30.39	31.31 31.92	32.59 32.90	30.16 30.46	31.89 31.43	32.82 32.70	2.5 1.5	*0.03* *0.01*
DLR	MAS CWI	32.11 31.02	32.92 31.83	33.65 32.76	32.30 31.01	33.04 31.97	33.56 32.85	32.32 31.03	32.91 31.99	33.60 32.85	32.36 31.15	32.86 32.12	33.49 33.12	2.3 1.2	*0.04* *0.03*
DLL	MAS CWI	30.28 30.08	31.41 31.64	32.94 32.73	30.58 29.89	32.19 31.32	33.36 32.74	30.15 30.19	31.51 31.54	32.89 32.83	30.07 29.98	31.35 30.95	32.60 32.53	2.1 1.4	*0.05* *0.05*

AAR, Anterior Arm Right; AAL, Anterior Arm Left; DLR, Dorsal Leg Right; DLL, Dorsal Leg Left; CWI, cold water immersion; MAS, massage; pre, before the Murph workout; postrec, immediately after the recovery intervention; post24, 24 h after the recovery intervention; post48, 48 h after the recovery intervention.

Participants in the CWI group displayed a consistent decrease in temperature asymmetry post-intervention. Initial measures showed an average temperature (50th percentile) of 32.12°C across all body sites, with a slight decrease to an average of 32.03°C at Post48. Notably, the greatest temperature reduction was observed immediately post-recovery (Postrec), with subsequent stabilizations at 24 and 48 hours. The MAS group showed a less pronounced but more sustained decrease in temperature across time points. Starting from an average of 32.07°C, temperatures slightly decreased and stabilized around 31.90°C from Postrec through to Post48.

Wilcoxon signed-rank tests were employed to assess the statistical significance of temperature changes from Pre to Postrec, Post24, and Post48. The analysis revealed that AAR MAS and DLR MAS groups demonstrated significant changes with Z-scores around 2.0 to 2.3 and p-values ranging from 0.04 to 0.05, indicating statistical significance in temperature reduction post-recovery.

CWI groups showed more significant results, particularly in AAL and DLL regions, with Z-scores lower (1.0 to 1.5) but p-values reaching as low as 0.01, suggesting a statistically significant impact of CWI on skin temperature.

These findings suggest that both interventions are effective in modifying skin temperature, with CWI showing a more immediate and significant effect, while MAS exhibits a more gradual and sustained effect.

This study investigated the efficacy of MAS and CWI on dynamic pressure algometry (PPT), musculoskeletal pain assessment (BPI), and perceived muscle soreness (VAS) across four time points: immediately before (Pre), immediately after (Postrec), 24 hours (Post24), and 48 hours (Post48) following a standard Murph workout (Table 3).

**Table 3:** Dynamic pressure algometry, musculoskeletal pain and perceived muscle soreness.

Variable	Group	Pre	Postrec	Post24	Post48	Time (p-value)	Intervention x time interaction (p-value)
**PPT**	MAS CWI	3.8 4.0	3.9 4.2	4.0 2.4	4.1 1.5	0.041 0.024	0.1632 0.0124
**BPI**	MAS CWI	1.34 1.42	3.99 3.12	3.40 3.54	3.90 1.30	0.0051 0.0058	0.1915 0.0159
**VAS**	MAS CWI	0 0.22	4.43 4.20	5.14 3.90	6.86 2.60	<0.0001 0.0057	0.1279 0.0169

PPT, Pressure pain threshold; Q-ADOM, Musculoskeletal Pain Assessment Questionnaire; BPI, Brief Pain Inventory; VAS, Visual Analog Scale; CWI, cold water immersion; MAS, massage; pre, before the Murph workout; postrec, immediately after the recovery intervention; post24, 24 h after the recovery intervention; post48, 48 h after the recovery intervention.

For the MAS group, PPT scores showed a progressive increase from Pre to Post48 (3.8 to 4.1), with significant changes over time (p = 0.041). The absence of a significant interaction effect (p = 0.1632) suggests that MAS consistently enhances the pain threshold regardless of the time point evaluated.

Conversely, the CWI group initially exhibited an increase in PPT scores, which subsequently decreased dramatically by Post48 (from 4.2 to 1.5). This decline was statistically significant over time (p = 0.024) and demonstrated a significant interaction between intervention type and time (p = 0.0124), indicating the effectiveness of CWI on pain thresholds over time.

Participants in the MAS group reported significant reductions in pain levels on BPI from Postrec to Post48 (p = 0.0051), with scores peaking at Postrec (3.99) and stabilizing by Post48 (3.90). No significant interaction was noted (p = 0.1915), suggesting similar pain reduction trajectories regardless of when measurements were taken.

The CWI group displayed significant variability in pain scores, peaking at Post24 (3.54) and dropping sharply by Post48 (1.30), with significant changes over time (p = 0.0058). Furthermore, the interaction effect was significant (p = 0.015), highlighting that the pattern of change in pain scores was distinctly influenced by the type of intervention across the observed time points.

The MAS group experienced a consistent increase in perceived muscle soreness across all time points at VAS, with significant escalation from Pre to Post48 (0 to 6.86, p < 0.0001). The interaction between time and intervention type was not significant (p = 0.1279), reflecting a uniform increase in soreness following the intervention.

Similarly, the CWI group reported an increase in perceived muscle soreness immediately post-workout, which then slightly decreased by Post48 (from 4.20 to 2.60, p = 0.0057). The interaction effect was significant (p = 0.0169), indicating that the patterns of change in soreness over time differed significantly between the interventions, suggesting a distinct response to CWI compared to MAS.

The analysis of pain intensity at rest revealed significant differences between the MAS and CWI groups at the baseline (Table 4). A substantial reduction in the prevalence of pain at rest was observed from pre-intervention to 48 hours post-intervention, particularly in the CWI group. Specifically, the CWI group showed a significant decrease in pain reports, from 87.5% at baseline to 0% at Post48 (p = 0.0154), indicating the effectiveness of CWI in managing pain. In contrast, the reduction in the MAS group was not statistically significant (p = NS).

**Table 4:** Pain characterization of volunteers according to the Q-ADOM.

Characteristic	All		MAS		CWI		p-value (Pre vs. Post48 within)	p-value (MAS vs. CWI at Pre)	p-value (MAS vs. CWI at Post48)
	Pre	Post48	Pre	Post48	Pre	Post48			
**In the last 4 weeks, did you experience pain in your bones, muscles, or joints AT REST?**									
**No**	10 (55.6%)	15 (83.3%)	3 (30%)	6 (60%)	7 (87.5%)	9 (100%)	MAS: NS	NS	0.0315
**Yes**	8 (44.4%)	3 (16.7%)	7 (70%)	4 (40%)	1 (12.5%)	0 (0%)	CWI: 0.0154		
**Location at rest?**									
**Head**	0 (0%)	0 (0%)	0 (0%)	0 (0%)	0 (0%)	0 (0%)	MAS: NS	NS	NS
**Trunk**	2 (11.1%)	1 (5.6%)	1 (10%)	1 (10%)	1 (12.5%)	0 (0%)	CWI: NS		
**Upper limbs**	4 (22.2%)	1 (5.6%)	2 (20%)	1 (10%)	2 (25%)	0 (0%)			
**Lower limbs**	2 (11.1%)	1 (5.6%)	2 (20%)	2 (20%)	0 (0%)	0 (0%)			
**Intensity of the most significant pain at rest**									
**None**	10 (55.6%)	15 (83.3%)	3 (30%)	6 (60%)	7 (87.5%)	9 (100%)	MAS: NS	NS	0.0467
**1-2 (mild)**	4 (22.2%)	2 (11.1%)	3 (30%)	3 (30%)	1 (12.5%)	0 (0%)	CWI: 0.0124		
**3-7 (moderate)**	3 (16.7%)	1 (5.6%)	3 (30%)	1 (10%)	0 (0%)	0 (0%)			
**8-10 (severe)**	1 (5.6%)	0 (0%)	1 (10%)	0 (0%)	0 (0%)	0 (0%)			
**In the last 4 weeks, did you experience pain in your bones, muscles, or joints DURING EXERCISE?**									
**No**	9 (50%)	15 (83.3%)	4 (40%)	6 (60%)	5 (62.5%)	9 (100%)	MAS: NS	NS	NS
**Yes**	9 (50%)	3 (16.7%)	6 (60%)	4 (40%)	3 (37.5%)	0 (0%)	CWI: 0.0264		
**Location during exercise?**									
**Head**	1 (5.6%)	0 (0%)	1 (10%)	0 (0%)	0 (0%)	0 (0%)	MAS: NS	NS	NS
**Trunk**	3 (16.7%)	1 (5.6%)	2 (20%)	1 (10%)	1 (12.5%)	0 (0%)	CWI: NS		
**Upper limbs**	2 (11.1%)	1 (5.6%)	2 (20%)	1 (10%)	0 (0%)	0 (0%)			
**Lower limbs**	3 (16.7%)	1 (5.6%)	1 (10%)	2 (20%)	2 (25%)	0 (0%)			
**Intensity of the most significant pain during exercise**									
**None**	9 (50%)	15 (83.3%)	4 (40%)	6 (60%)	5 (62.5%)	9 (100%)	MAS: NS	NS	0.0381
**1-2 (mild)**	5 (27.8%)	2 (11.1%)	4 (40%)	3 (30%)	1 (12.5%)	0 (0%)	CWI: 0.0334		
**3-7 (moderate)**	3 (16.7%)	1 (5.6%)	2 (20%)	1 (10%)	1 (12.5%)	0 (0%)			
**8-10 (severe)**	1 (5.6%)	0 (0%)	0 (0%)	0 (0%)	1 (12.5%)	0 (0%)			

Note: *Statistically significant difference (Pre vs. Post48 within: This is calculated using McNemar’s test to show changes within each group over time;

MAS vs. CWI at Pre and MAS vs. CWI at Post48: These are calculated using Fisher’s Exact Test to compare differences between groups at each time point., p ≤ 0.05).

Only those who answered “yes” to the previous question were included.

While the changes in pain intensity at rest were notable, they were significant only in the CWI group, with no participants experiencing moderate or severe pain at Post48 (p = 0.0124). No significant changes were observed in the location of pain at rest for either group.

The CWI group demonstrated a significant reduction of pain during exercise by Post48, decreasing from initial reports to no reports (p = 0.0264). This contrasts with the MAS group, which, while showing a reduction, did not reach statistical significance. No significant changes were observed in the location of pain during exercise for either group.

When comparing the two groups, there were no statistically significant differences in pain prevalence or intensity at baseline (p = NS for all comparisons). However, at Post48, the CWI group exhibited significantly better outcomes than the MAS group in reducing the prevalence and severity of pain (p < 0.05), underscoring the superior efficacy of CWI in pain management during and after physical activities.

## Discussion

The results of this study highlight the differential impacts of CWI and MAS on pain management and recovery following the CrossFit® Murph workout. While CWI demonstrated statistically significant reductions in pain prevalence both at rest and during exercise, the MAS group also showed a positive trend in pain reduction and improved PPT, despite these effects not reaching statistical significance. Even though the effects of MAS were less robust compared to CWI, the trend toward improvement may hold clinical significance, especially for practitioners looking for alternatives to CWI. This underscores the importance of considering both statistical and clinical relevance when evaluating recovery interventions, as these findings may influence clinical decision-making in contexts where MAS is more feasible or preferable.

The analysis revealed a significant reduction in pain in the CWI group, with no pain reported by participants at 48 hours post-intervention, indicating a substantial and sustained impact on muscle soreness. Conversely, while MAS participants did not experience the same immediate relief, a gradual improvement in PPT scores over time suggests a cumulative benefit, potentially enhancing pain tolerance in the longer term. While CWI was more effective at rapidly reducing pain intensity, the MAS group showed a more sustained and gradual improvement, which may be valuable in managing chronic muscle discomfort. These subtle differences in recovery dynamics should be considered when selecting a recovery modality based on the specific needs of athletes.

Regarding the mechanistic explanations of these interventions, both CWI and MAS are believed to reduce muscle soreness and enhance recovery through distinct physiological mechanisms. CWI, by inducing vasoconstriction, reducing metabolic activity, and decreasing inflammation, likely contributes to the rapid reduction in pain and soreness immediately after exercise. This is supported by prior studies suggesting that cold therapies can alleviate muscle swelling and reduce pain perception through cold-induced analgesia and decreased nerve conduction speed [20,21]. The prolonged effects observed in our study, with pain reduction lasting up to 48 hours post-intervention, align with these proposed mechanisms and suggest that CWI may offer extended recovery benefits beyond the immediate post-exercise phase.

On the other hand, MAS primarily operates through mechanical pressure that improves circulation, reduces muscle tension, and enhances parasympathetic activity. While the effects of MAS on pain reduction were more gradual and less pronounced, the improvement in PPT scores over time suggests that regular massage therapy may contribute to long-term pain management and muscle recovery, especially in athletes with ongoing muscle stiffness or those recovering from repetitive exercise [6,22]. This cumulative effect highlights the potential utility of MAS in the context of chronic conditions and recovery from intense physical activities.

Interestingly, inter-individual variability in response to the recovery interventions was evident, with some participants showing greater pain relief from CWI while others benefitted more from MAS. This variability may be influenced by factors such as baseline pain sensitivity, psychological readiness, and athletic training history. For example, athletes with a history of chronic muscle soreness may respond differently to the two recovery methods than those with less frequent exposure to intense physical exertion [21,23]. This variability supports the notion that personalized recovery strategies are crucial for optimizing recovery outcomes in athletes, rather than adopting a one-size-fits-all approach.

The results also point to potential interaction effects between sex and recovery type, as CWI may induce more complex neurophysiological responses, such as nociceptive sensitization followed by a rebound effect in pain perception. However, we did not analyze these potential interactions in detail in this study, and future research should explore these aspects further to clarify the relationship between recovery interventions and individual characteristics [24].

While CWI and MAS showed significant effects in reducing pain and soreness, there are important limitations to consider. The lack of a control group and reliance on self-reported outcomes introduce potential biases. For example, participants’ expectations about the effectiveness of the interventions might have influenced their pain reports, particularly given that they were aware of their assigned intervention. Moreover, the small sample size limits the generalizability of the findings, and future studies should aim to include larger, more diverse cohorts. Additionally, while subjective pain assessments like VAS and BPI are widely used, they are inherently prone to individual reporting bias, and objective biomarkers (e.g., blood markers or muscle enzyme levels) would strengthen the findings [21,25]. However, due to logistical constraints, these objective measures were not included in this study. The use of self-reported questionnaires and PPT is a limitation, as these outcomes are still subjective and may not fully capture the physiological processes underlying recovery.

The combination of CWI and MAS as complementary recovery strategies is an intriguing area for future research. Some studies have suggested that combining cold immersion with other techniques might provide synergistic effects, enhancing recovery beyond what each intervention could achieve alone [14]. Future studies should explore the potential benefits of combining these two interventions to optimize recovery strategies for athletes, particularly in high-intensity exercise contexts like CrossFit®.

In conclusion, while CWI showed more immediate and statistically significant effects on pain and soreness, MAS demonstrated a consistent trend toward improvement in PPT and long-term pain tolerance. Both interventions are effective in enhancing recovery, with their benefits depending on the athlete’s specific needs and preferences. Future studies should explore the mechanisms of these interventions in more detail, incorporate objective biomarkers, and consider blinding techniques to reduce bias, while addressing the limitations of sample size and lack of control groups to enhance the reliability of findings.

## Conclusion

In conclusion, our study confirms the effectiveness of both CWI and MAS in managing post-exercise pain, with CWI showing a more immediate and pronounced effect on reducing pain and soreness. Future research should explore the optimal timing and combinations of these interventions to maximize recovery outcomes and adapt them to individual athlete needs. The integration of these recovery strategies into sports rehabilitation programs should consider these differential effects to tailor interventions that best meet the athletes’ recovery and performance goals.

## Supporting information

S1 File CONSORT checklist of information. This table presents the CONSORT (Consolidated Standards of Reporting Trials) 2010 checklist items as applied to this study protocol.
(DOC)

S2 File Trial study protocol (en). Study protocol for the randomized controlled trial evaluating cryotherapy in delayed onset muscle soreness (DOMS) – English version.
(PDF)

S3 File Trial study protocol (pt). Study protocol for the randomized controlled trial evaluating cryotherapy in delayed onset muscle soreness (DOMS) – Portuguese version.
(PDF)
