# Supplementary material for: Effectiveness of cold-water immersion vs. massage in reducing delayed-onset muscle soreness and enhancing recovery following CrossFit® Murph Workout: Randomized rial
Source: PLoS One. 2025 Aug 13;20(8):e0329892. doi: 10.1371/journal.pone.0329892 (PMC12349088; doi:10.1371/journal.pone.0329892)
Supplement: S1 File — This table presents the CONSORT (Consolidated Standards of Reporting Trials) 2010 checklist items as applied to this study protocol. (DOC) [file pone.0329892.s001.doc]

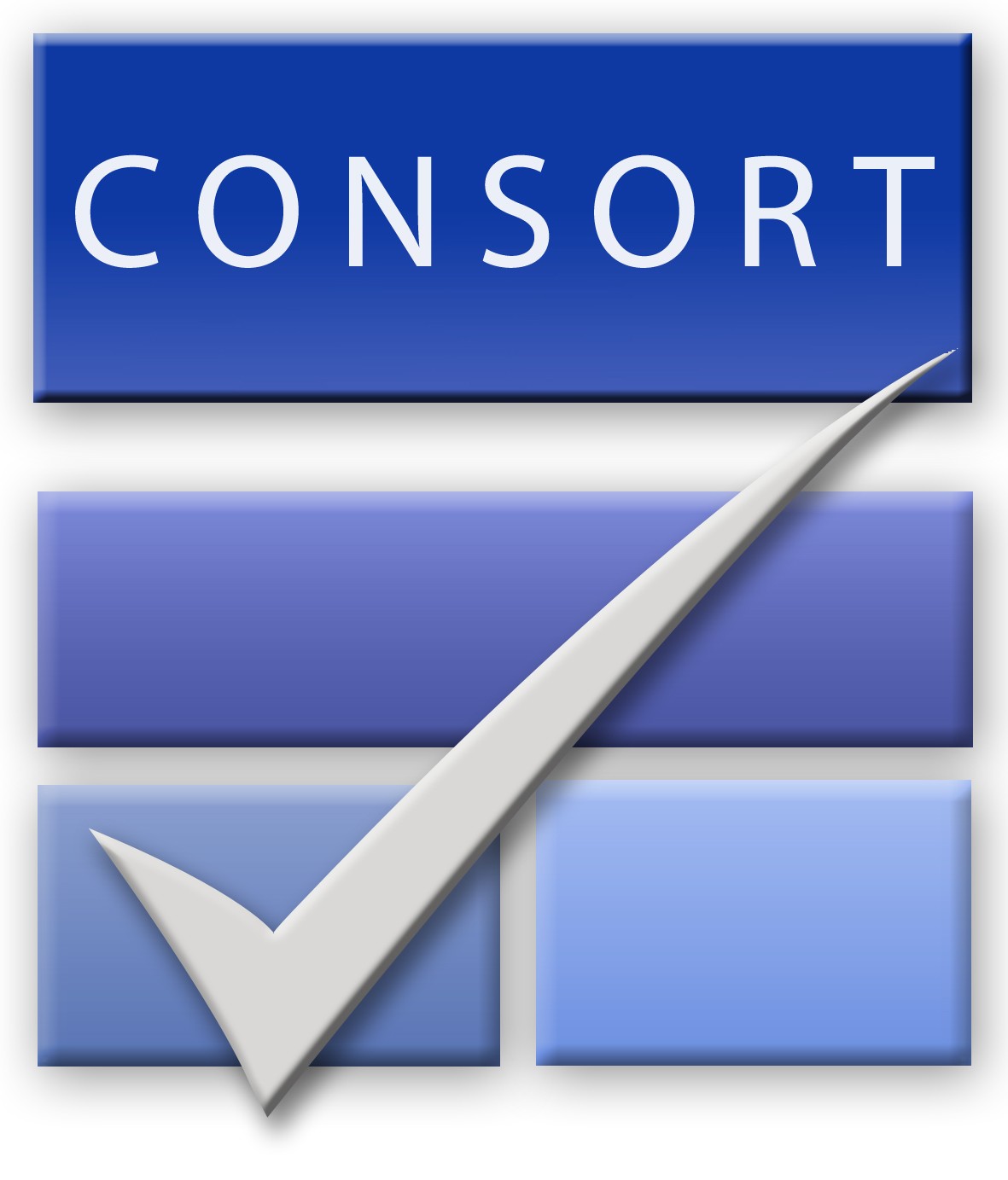
CONSORT 2010 checklist of information to include when reporting a randomised trial*

| Section/Topic | Item No | Checklist item | Reported on page No |
| --- | --- | --- | --- |
| Title and abstract | | | |
|  | 1a | Identification as a randomised trial in the title | Page 1, line4 |
|  | 1b | Structured summary of trial design, methods, results, and conclusions (for specific guidance see CONSORT for abstracts) | Page 2, Line 15 |
| Introduction | | | |
| Background and objectives | 2a | Scientific background and explanation of rationale | Page 3, Line 42 |
|  | 2b | Specific objectives or hypotheses | Page 4, Line 70 |
| Methods | | | |
| Trial design | 3a | Description of trial design (such as parallel, factorial) including allocation ratio | Page 5, Line 130 |
|  | 3b | Important changes to methods after trial commencement (such as eligibility criteria), with reasons | Page 4, Line 77 |
| Participants | 4a | Eligibility criteria for participants | Page 4, Line 77 |
|  | 4b | Settings and locations where the data were collected | Page 4, Line 77 |
| Interventions | 5 | The interventions for each group with sufficient details to allow replication, including how and when they were actually administered | Page 5. Line 146 |
| Outcomes | 6a | Completely defined pre-specified primary and secondary outcome measures, including how and when they were assessed | Page 5, Line 130 |
|  | 6b | Any changes to trial outcomes after the trial commenced, with reasons | Page 3, Line 91 |
| Sample size | 7a | How sample size was determined | Page 7, Line 160 |
|  | 7b | When applicable, explanation of any interim analyses and stopping guidelines | Page 7, Line 168 |
| Randomisation: |  |  | Page 7, Line 146 |
| Sequence generation | 8a | Method used to generate the random allocation sequence |  |
|  | 8b | Type of randomisation; details of any restriction (such as blocking and block size) | Page 7, Line 146 |
| Allocation concealment mechanism | 9 | Mechanism used to implement the random allocation sequence (such as sequentially numbered containers), describing any steps taken to conceal the sequence until interventions were assigned | Page 7, Line 146 |
| Implementation | 10 | Who generated the random allocation sequence, who enrolled participants, and who assigned participants to interventions | Page 7, Line 146 |
| Blinding | 11a | If done, who was blinded after assignment to interventions (for example, participants, care providers, those assessing outcomes) and how |  |
|  | 11b | If relevant, description of the similarity of interventions |  |
| Statistical methods | 12a | Statistical methods used to compare groups for primary and secondary outcomes | Page 7, Line 168 |
|  | 12b | Methods for additional analyses, such as subgroup analyses and adjusted analyses | Page 7, Line 168 |
| Results | | | |
| Participant flow (a diagram is strongly recommended) | 13a | For each group, the numbers of participants who were randomly assigned, received intended treatment, and were analysed for the primary outcome | Page 8, Line 177 |
|  | 13b | For each group, losses and exclusions after randomisation, together with reasons | Page 8, Line 177 |
| Recruitment | 14a | Dates defining the periods of recruitment and follow-up | Page 8, Line 177 |
|  | 14b | Why the trial ended or was stopped | Page 8, Line 177 |
| Baseline data | 15 | A table showing baseline demographic and clinical characteristics for each group | Page 8, Line 190 |
| Numbers analysed | 16 | For each group, number of participants (denominator) included in each analysis and whether the analysis was by original assigned groups | Page 8, Line 190 |
| Outcomes and estimation | 17a | For each primary and secondary outcome, results for each group, and the estimated effect size and its precision (such as 95% confidence interval) | Page 9, Line 219 |
|  | 17b | For binary outcomes, presentation of both absolute and relative effect sizes is recommended |  |
| Ancillary analyses | 18 | Results of any other analyses performed, including subgroup analyses and adjusted analyses, distinguishing pre-specified from exploratory | Page 11, Line 254 |
| Harms | 19 | All important harms or unintended effects in each group (for specific guidance see CONSORT for harms) |  |
| Discussion | | | |
| Limitations | 20 | Trial limitations, addressing sources of potential bias, imprecision, and, if relevant, multiplicity of analyses | Page 15, Line 335 |
| Generalisability | 21 | Generalisability (external validity, applicability) of the trial findings | Page 14, Line 287 |
| Interpretation | 22 | Interpretation consistent with results, balancing benefits and harms, and considering other relevant evidence | Page 14, Line 287 |
| Other information | | |  |
| Registration | 23 | Registration number and name of trial registry | Page 4, Line 90 |
| Protocol | 24 | Where the full trial protocol can be accessed, if available | Page 4, Line 90 |
| Funding | 25 | Sources of funding and other support (such as supply of drugs), role of funders |  |

Citation: Schulz KF, Altman DG, Moher D, for the CONSORT Group. CONSORT 2010 Statement: updated guidelines for reporting parallel group randomised trials. BMC Medicine. 2010;8:18.
© 2010 Schulz et al. This is an Open Access article distributed under the terms of the Creative Commons Attribution License (<http://creativecommons.org/licenses/by/2.0>), which permits unrestricted use, distribution, and reproduction in any medium, provided the original work is properly cited.

*We strongly recommend reading this statement in conjunction with the CONSORT 2010 Explanation and Elaboration for important clarifications on all the items. If relevant, we also recommend reading CONSORT extensions for cluster randomised trials, non-inferiority and equivalence trials, non-pharmacological treatments, herbal interventions, and pragmatic trials. Additional extensions are forthcoming: for those and for up-to-date references relevant to this checklist, see [www.consort-statement.org](http://www.consort-statement.org/).
