# Supplementary material for: Effectiveness of cold-water immersion vs. massage in reducing delayed-onset muscle soreness and enhancing recovery following CrossFit® Murph Workout: Randomized rial
Source: PLoS One. 2025 Aug 13;20(8):e0329892. doi: 10.1371/journal.pone.0329892 (PMC12349088; doi:10.1371/journal.pone.0329892)
Supplement: S2 File — Study protocol for the randomized controlled trial evaluating cryotherapy in delayed onset muscle soreness (DOMS) – English version. (PDF) [file pone.0329892.s002.pdf]

Machine Translated by Google

MINISTRY OF HEALTH - National Health Council - National Research Ethics Committee – CONEP

RESEARCH PROJECT INVOLVING HUMAN BEINGS

Research Project:
EVALUATION OF THE EFFECTS OF CRYOTHERAPY ON RECOVERY FROM DELAYED ONSET MUSCLE SORENES IN ATHLETES AFTER INTERVAL TRAINING HIGH INTENSITY IN WOD MURPH IN CROSSFIT: A RANDOMIZED, CONTROLLED CLINICAL TRIAL

Preliminary Information

Main Responsible

CPF/Document:	Name: Marcelo Lourenco da Silva
Phone: 1634215280	Email: marcelo.lourenco@unifal-mg.edu.br

Proposing Institution

CNPJ: 17,879,859/0001-15	Name of Institution: FEDERAL UNIVERSITY OF ALFENAS - UNIFAL-MG

Is it an international study? No

Research Team

CPF/Document	Name
	Gabrielly Santos Pereira
	Josie Resende Torres da Silva

Study Area

Major Areas of Knowledge

- Major Area 4. Health Sciences

Main Purpose of the Study

- Clinical

Public Title of Research: EVALUATION OF THE EFFECTS OF CRYOTHERAPY ON RECOVERY FROM DELAYED ONSET MUSCLE SORENES IN ATHLETES AFTER HIGH INTENSITY INTERVAL TRAINING IN THE MURPH WOD IN CROSSFIT: REHEARSAL CLINICAL, CONTROLLED AND RANDOMIZED

Public Contact

CPF/Document	Name	Telephone	E-mail
220.945.588-00	Marcelo Lourenco da Silva	1634215280	marcelo.lourenco@unifal-mg.edu.br

Scientific Contact: Marcelo Lourenco da Silva

Machine Translated by Google

Study Design / Financial Support

Study Design: Intervention/Experimental

Health conditions or problems

Health Condition or Problem
Delayed onset muscle soreness

General Descriptors for Health Conditions

ICD1-10: International Classification of Diseases

ICD Code	CID Description
M62	Other muscle disorders

Specific Descriptors for Conditions of

ICD1-10: International Classification of Diseases

ICD Code	CID Description
M62	Other muscle disorders

Type of Intervention: Experimental

Nature of Intervention

- Other Cryotherapy

Intervention Descriptors

Intervention Descriptors

Interventions
Cryotherapy

Phase

- Phase 1

There will be use of placebo or the existence of groups that will not undergo any intervention: The use of a placebo allows a more accurate comparison between the effects of real cryotherapy and the psychosomatic responses of the participants, ensuring that any improvements observed in the intervention group are truly attributed to cryotherapy and not to the placebo effect.

Design:

Initially, we will collect baseline data from all participating athletes so that we can create a baseline. We will then use the musculoskeletal pain assessment questionnaire (E-ADOM) to assess participants' level of muscle discomfort prior to the start of training. We will then administer the Brief Pain Inventory (BPI), a tool that allows us to measure pain intensity and its impact on individuals' daily activities. In addition, the Visual Analogue Scale (VAS) will be used so that participants can indicate pain intensity on a scale of 0 to 10, providing a subjective measure of pain.

For a more objective assessment, we will use algometry, which measures pain sensitivity by applying controlled pressure to the gastrocnemius muscle. In addition, we will use thermographic imaging to observe the temperature of the gastrocnemius muscle, which may indicate inflammation or other physiological processes. Finally, we will collect blood samples to measure levels of creatine kinase (CK), an enzyme that serves as a marker of muscle damage. Analyzing these levels will provide us with important information about the degree of muscle damage caused by training.

After collecting baseline data, athletes will perform the WOD Murph, which consists of running 1 mile, performing 100 pull-ups, 200 push-ups, 300 air squats, and running another 1 mile, an intense physical activity that generates a DOMS.

After completing the training, we will perform an intervention with immersion cryotherapy. An ice bath will be provided, in which the athlete will enter covering the entire gastrocnemius muscle. The application of ice to the participants' muscles aims to reduce inflammation and pain, promoting faster recovery.

To evaluate the effects of the cryotherapy intervention, we will repeat the collection of all data collected pre-workout at 24, 48 and 72 hours after training for possible conclusions.

Financial Support

CNPJ	Name	E-mail	Telephone	Type
				Financing Own

Keyword

Machine Translated by Google

Keyword
high intensity interval training
delayed onset muscle soreness
cryotherapy

Study Details

Summary:

Currently, more than 30% of the world's population is physically inactive, and one of the main reasons is time constraints. This fact has significant implications for global health, suggesting recommendations for shorter and more effective exercises, such as high-intensity interval training (HIIT). This type of training has the ability to improve aerobic fitness, body composition, and cardiometabolic health. An example of HIIT is the CrossFit® "Murph" workout, which combines aerobic and resistance modalities in intense circuits. The CrossFit "Murph" workout, one of the most challenging, often results in delayed onset muscle soreness (DOMS). Thus, ice immersion (cryotherapy) stands out as a viable therapeutic modality to study, due to its anti-inflammatory and analgesic effects promoting rapid recovery after exercise. Therefore, the present study aims to analyze the effect of cryotherapy as an intervention in the recovery of DOMS after practicing the HIIT of the CrossFit® Murph workout of the day (WOD). This study is a randomized controlled clinical trial, in which participants are divided into control (CG) and intervention (IG) groups. Assessment instruments include the visual analogue pain scale (VAS), BORG scale, brief pain inventory (BPI), questionnaire for assessing musculoskeletal pain in exercise practitioners (Q-ADOM), algometer, thermographic camera and collection of blood samples to assess creatine kinase CK. The results are expected to contribute to the development of more precise intervention protocols with cryotherapy in DOMS.

Introduction:

More than 30% of the world's population is physically inactive, which has significant implications for global health (Hallal et al., 2012). There is now growing recognition that the time constraints faced by many people when initiating exercise programs make previous recommendations unfeasible, suggesting that shorter intervals of time may be more appropriate (Molanorouzi et al., 2015). Furthermore, high-intensity exercise has been shown to provide improved outcomes in terms of strength and cardiorespiratory fitness (Garber et al., 2011). High-intensity interval training (HIIT) is generally defined as the repetition of short or long bursts of intense exercise, interspersed with periods of active or passive recovery. However, there is no consensus on the specificity, frequency, and duration of exercise required to maximize health benefits and prevent diseases associated with a sedentary lifestyle (Gillen and Gibala, 2014). This type of training can improve aerobic fitness, body composition, and cardiometabolic health in diverse populations (Babraj et al., 2009). HIIT training is generally recommended to be performed with full-body exercises and cyclic movements, such as walking, running, swimming, or cycling, which do not significantly increase muscle strength or power (Waller et al., 2011). Multimodal training combines several modalities, such as aerobic endurance, strength, and circuit training. There is some evidence that continuous circuit training can induce a cardiovascular response, but the intensity used generally varies from low to moderate (Hunter et al., 2003). According to McRae et al. (McRae et al., 2012), there is limited evidence that low-volume HIIT may be as effective as continuous aerobic training in improving aerobic capacity and superior muscular endurance gains, although this method has not yet been widely investigated. An example of HIIT is CrossFit®, which is a high-intensity, multimodal workout that incorporates functional (multi-joint) movement patterns performed in a circuit format, with short pauses or rest periods between exercises or sets of exercises (Bergeron et al., 2011). Since 2013, HIIT has been one of the three largest global fitness trends, with CrossFit® being a major contributor to this popularity, according to the ACSM's annual survey (Thompson, 2016). According to Wood (Wood et al., 2022), CrossFit® is effective in improving cardiovascular fitness and body composition in exercisers of all fitness levels. However, the CrossFit® methodology has also faced criticism for not strictly following established training principles, and there is little research on the specific responses to different types of training within this methodology. The International Association for the Study of Pain (IASP) defines pain as an unpleasant sensory and emotional experience associated with, or similar to, actual or potential tissue injury (DeSantana et al., 2020). In this context, delayed onset muscle soreness (DOMS) consists of the sensation of pain or discomfort in the muscles that typically increases in intensity in the first 24 hours and peaks between 24 and 48 hours after exercise (Cheung et al., 2003). This type of pain is a signal that the muscles are adapting in response to intense physical activities, especially those that involve eccentric contractions, such as the lowering phase of squats, weightlifting in strength training, or downhill running. During these activities, blood levels of creatine kinase (CK) increase, indicating muscle damage. A notable example of a workout that can induce DOMS is the "Murph," one of CrossFit's most iconic workouts, known for its physical and mental demands. Created in memory of Lieutenant Michael P. Murphy, a US Navy SEAL killed in action, this Workout of the Day (WOD) consists of running 1 mile (1.6 km), performing 100 pull-ups, 200 push-ups, 300 air squats, and running another 1 mile (1.6 km). The goal is to complete the workout in the shortest possible time, and it is a high-intensity aerobic exercise that is influenced by anaerobic performance and the recovery capacity of high-intensity exercise (Carreker and Grosicki, 2020). Due to its intensity and high volume of exercise, Murph can induce DOMS, often making subsequent workouts difficult due to discomfort. Analyzing the occurrence of DOMS and its effects after the Murph WOD, a high-intensity activity, is essential. Recovery (known as recovery) for DOMS, immediately after training as an intervention measure, should be considered, thus improving strategies for recovery of athletes. Our project aims to understand how ice immersion applied immediately after the practice of this intense activity can minimize DOMS. Ice immersion (cryotherapy) stands out as a viable therapeutic modality to study. Several studies indicate that cryotherapy can reduce inflammation and delayed onset muscle soreness, promoting faster recovery after HIIT. For example, a study conducted by Leeder et al. (Leeder et al., 2012) demonstrated that cold water immersion can reduce delayed onset muscle soreness and accelerate muscle recovery . Furthermore, cryotherapy is widely used and accepted in sports settings, and there is a significant body of research supporting its efficacy (Alexander et al., 2021). Therefore, investigating ice immersion may provide relevant and applicable data to improve athletes' recovery after high-intensity activities such as WOD Murph. Thus, this scientific project aims to fill gaps in knowledge about DOMS after performing HIIT in WOD Murph in CrossFit, also verifying whether pain persists with the intervention. It is expected that the results of these investigations will contribute to the development of more effective therapeutic protocols and significant improvements in the training quality of athletes affected by this painful condition.

Hypothesis:

The hypothesis of the study is that the application of immersion cryotherapy after performing the intense CrossFit® "Murph" workout will significantly reduce delayed onset muscle soreness (DOMS) and inflammation, promoting faster and more efficient recovery in

Machine Translated by Google

athletes, compared to those who do not receive cryotherapy intervention.

Primary Objective: To

investigate the effects of cryotherapy as a recovery intervention on the manifestation of DOMS in athletes after performing intense physical activities in CrossFit.

Secondary Objective: To

verify the intensity of pain perceived in DOMS after intense training, Murph, using the algometer as a research method. To verify if DOMS alters the analgesic perception in the VAS and BPI after Murph training. To verify if DOMS alters the perception of musculoskeletal pain with the Q-ADOM questionnaire after Murph training. To evaluate the levels of protein kinase CK as a biomarker of DOMS after Murph training. To verify through thermography if there is a change in muscle temperature before and after training.

Proposed Methodology:

Proposed Methodology: Study Design: This study will be a randomized, controlled clinical trial designed to evaluate the efficacy of cryotherapy in the recovery of delayed onset muscle soreness (DOMS) in CrossFit athletes after completing the Murph workout. Study Location and Population: The study will be conducted at the Capixaba training center, with support from the Laboratory of Neuroscience, Neuromodulation and Pain Study (LANNED), located in Alfenas, Minas Gerais. The study population will consist of 30 athletes who participate in training at the Capixaba box. Sample: Thirty athletes who participated in the Murph training at the Capixaba box will be selected. Participants will be recruited through invitations made directly at the Capixaba box, where the athletes usually train regularly. Brief presentations will be given before or after the training sessions to explain the objectives and importance of the study, as well as the inclusion and exclusion criteria. In addition, informational pamphlets will be distributed and posters will be posted around the box, containing details about the study and contact information for the researchers. Individuals will be divided into 2 groups: the control group and the intervention group, allocated through a randomization process performed using the Research Randomizer website. Sample Randomization: Volunteers will be divided into CG and IG through randomization, using the Research Randomizer website to ensure random allocation of participants. Sample Calculation: The sample calculation will be performed according to Cariappa (2006), estimating 15 volunteers per group to ensure a statistical power of 85% (ß=0.20) and a significance level of $p<0.05$. Assessment Instruments: Several instruments will be used to assess the pain and recovery of participants: 1.

Assessment Questionnaire for Application of the Protocol: Collects information on supplementation, diet, training frequency, use of therapeutic modalities and sleep quality. 2. Visual Analog Scale (VAS): Assesses pain intensity, graded from 0 to 10. 3. BORG: Assesses the perception of physical effort, using a scale from 0 to 10. 4. Brief Pain Inventory Scale (BPI): Assesses pain intensity and its impact on daily activities. 5. Questionnaire for the Assessment of Musculoskeletal Pain in Exercise Practitioners (Q-ADOM): Identifies the characteristics of pain and its influence on the practice of physical exercise. 6. Algometer: Measures sensitivity to pain by applying controlled pressure. 7. Thermographic Camera: Detects infrared radiation emitted by the body, visualizing the temperature of the skin surface. 8.
CK Protein Quantification: Measurement of creatine kinase (CK) levels in blood as a marker of muscle damage. Intervention: The intervention will consist of cold water immersion sessions (cryotherapy) immediately following the Murph workout. The water temperature will be kept below 15°C and the duration of the immersion will be 10 minutes. The cryotherapy session will involve immersing the athletes' legs in a suitable container, ensuring that all major muscle areas involved in the exercise are treated. Experimental Procedures: Initially, baseline data will be collected using E-ADOM, BPI, VAS, algometry, thermography and blood samples to measure CK. After baseline data collection, athletes will perform the Murph WOD. After the workout, the cryotherapy intervention will be performed. Data will be collected again 24, 48 and 72 hours post-workout to assess the effects of the intervention on DOMS.

Inclusion Criteria:

Participants must be between 18 and 45 years of age and have at least 6 months of regular CrossFit training experience, training at least three times per week. It is essential that athletes be considered healthy and have no current or chronic musculoskeletal injuries that could interfere with the performance of the Murph WOD or the application of cryotherapy. Additionally, participants must not have performed the "Murph" WOD or similar intensity exercises in the four weeks prior to the start of the study.

All participants must provide written informed consent indicating that they understand the study procedures, risks, and benefits. They must also be available for training sessions, cryotherapy application, and subsequent assessments within the time frame established by the study. Athletes' body mass index (BMI) must be between 18.5 and 30 kg/m² to exclude extremes of body mass that could influence the results. Additionally, participants must not have used cryotherapy regularly as a recovery method in the three months prior to the study. It is important that athletes demonstrate the ability to follow study instructions and protocols consistently and correctly. Finally, participants must be able to read and understand the language in which the study and informed consent are presented, ensuring that they understand all study instructions and requirements.

Exclusion Criteria:

Participants will be excluded if they have any medical condition that could be exacerbated by intense exercise or cold exposure, such as severe cardiovascular, respiratory or metabolic disease. Athletes with a history of chronic or recent musculoskeletal injuries that could interfere with the performance of the Murph WOD or the application of cryotherapy will also be excluded.

In addition, individuals who have performed the WOD Murph or similar intensity exercises in the four weeks prior to the start of the study will be excluded to avoid effects of previous training. Participants who have used cryotherapy regularly as a recovery method in the three months prior to the study will also not be included to ensure that the intervention is novel to all subjects and its effects can be more clearly assessed.

Athletes who are unable to provide written informed consent or who do not fully understand the study procedures, risks and benefits will be excluded. Likewise, individuals who are not available to participate in all training sessions, cryotherapy application and subsequent assessments within the established study period will be disregarded. Those who present a body mass index (BMI) outside the range of 18.5 to 30 kg/m², to exclude extremes of body mass that could influence the results, will also be excluded.

Participants who do not demonstrate the ability to follow study instructions and protocols consistently and correctly will be excluded to ensure uniformity in the application of recovery methods. Individuals who cannot read and understand the language in which the study and informed consent are presented will be excluded, ensuring that all participants fully understand the instructions and requirements of the study. In addition, athletes who are using specific dietary supplements for muscle recovery or anti-inflammatories will be excluded, as these substances may interfere with the results of the research.

Machine Translated by Google

Risks:

Cryotherapy, although widely used and generally safe, may present some potential risks. Exposure to extremely low temperatures may cause temporary discomfort, including a sensation of intense cold, tingling, or numbness of the skin. In rare cases, immersion in cold water may result in skin injuries such as frostbite or frostbite if the temperature is not properly monitored. Individuals with pre-existing medical conditions, such as cardiovascular, respiratory, or metabolic problems, may be at greater risk of adverse complications due to vasoconstriction and systemic response to cold. Therefore, prior medical evaluation will be performed to identify and exclude such individuals.

Participants may also experience a temporary increase in muscle stiffness or discomfort after cold water immersion, especially if they are not accustomed to this type of therapy. Additionally, blood collection to measure creatine kinase (CK) levels may cause mild discomfort, bruising, or infection at the venipuncture site, although these risks are minimal when proper aseptic procedures are followed.

Benefits:

The benefits of participating in the study include the potential for reduced DOMS and improved muscle recovery, allowing athletes to return to their physical activities more quickly and with less discomfort. Participants will have access to a potentially effective therapeutic intervention that may not be available to them outside of the study context. Additionally, the data obtained may provide valuable insights into the efficacy of cryotherapy, contributing to the development of more effective recovery practices in the field of CrossFit and sports in general.

Participants can also benefit from more detailed monitoring of their fitness and response to exercise, with feedback and guidance from qualified professionals. This monitoring can help them better understand how their body responds to intense training and recovery strategies, potentially improving their exercise performance and preventing future injuries.

In terms of scientific contribution, the results of the study can benefit the wider sports community by providing evidence that can guide the implementation of effective recovery strategies for other athletes.
As a risk-minimizing measure, all participants will first undergo a pre-medical evaluation to identify possible contraindications for cryotherapy. This evaluation will include a detailed physical examination and a review of medical history to exclude individuals with cardiovascular, respiratory or metabolic conditions that may be exacerbated by cold exposure.

During cryotherapy sessions, the water temperature will be strictly monitored and kept below 15°C, using calibrated thermometers to ensure accuracy. Immersion will be limited to a maximum of 10 minutes to avoid any damage to the skin or underlying tissues.
Furthermore, supervision by qualified professionals throughout the intervention will ensure that any discomfort or adverse effects are immediately identified and treated. Participants will be instructed to report any abnormal sensations or discomforts during the immersion, allowing immediate interruption of the session if necessary.

To minimize the risks associated with blood collection for measurement of creatine kinase (CK) levels, strict aseptic procedures will be followed. Collection will be performed by trained professionals using sterile materials to reduce the risk of infection. In addition, puncture sites will be carefully selected and monitored to prevent bruising and other minor complications.

Participants will receive detailed instructions on study procedures and safety measures before the intervention begins.
These instructions include guidance on the importance of proper hydration, warming up and cooling down before and after workouts and the cryotherapy session. Information will also be provided on how to monitor and report any adverse effects following the sessions.
In addition to immediate safety measures, a continuous monitoring system will be established to monitor participants throughout the study. This will include regular assessments of muscle pain and other symptoms using standardized methods such as the Visual Analogue Scale (VAS), the Brief Pain Inventory (BPI) and the Questionnaire for Assessment of Musculoskeletal Pain in Exercisers (Q-ADOM). The use of algometers and thermal imaging cameras will provide objective assessments of pain and inflammation, ensuring that any significant changes in participants' condition are detected early.

In the event of any adverse event or complication, participants will have immediate access to appropriate medical care. Emergency protocols will be established and professionals involved in the study will be prepared to deal with any unexpected situation.

Data Analysis Methodology: Initially,

descriptive analyses will be performed to summarize the demographic and clinical characteristics of the participants, as well as the results of the pain measurements. To compare pain levels before and after the cryotherapy intervention, paired t-tests or repeated measures analysis of variance (ANOVA) will be used, depending on the normality of the data. If the data do not follow a normal distribution, nonparametric tests such as the Wilcoxon test will be applied.

Additionally, multiple regression analyses may be performed to identify potential factors that influence the efficacy of cryotherapy, such as age, gender, CrossFit experience, and training intensity level. Correlation analyses will be conducted to investigate the relationships between different pain assessment methods and CK levels. Statistical significance will be set at a level of $p < 0.05$. All data will be analyzed using SPSS 20 statistical software.

Primary Outcome:

This research is expected to contribute to more accurate protocols and assessment methods for DOMS. It will provide coaches, physiotherapists, and athletes with concrete data on how to optimize post-exercise recovery, improving performance and adherence to training programs. It will also fill a significant gap in the literature regarding the effectiveness of post-exercise recovery strategies.

Sample Size in 30

Date of First Recruitment: 08/11/2024

Recruitment Countries

Country of Origin of the Study	Country	No. of survey participants
Yes	BRAZIL	30

Machine Translated by Google

Other Information

Will there be use of secondary data sources (medical records, demographic data, etc.)?

No

Report the number of individuals approached personally, recruited, or who will undergo some type of intervention at this center search:

30

Groups into which research participants will be divided at this center

Group ID	No. of Individuals	Interventions to be carried out
Control	15	They will not do cryotherapy
Cryotherapy	15	Cryotherapy

Is the Study Multicenter in Brazil?

No

Do you propose waiving the TCLE?

No

Will samples be retained for bank storage?

Yes

Justification:

Yes, samples will be retained for bank storage. Blood samples collected to measure creatine kinase (CK) levels will be stored in a biological sample bank. This storage will allow for future additional analyses that may arise as relevant to the study or to subsequent research related to delayed onset muscle soreness (DOMS) and muscle recovery. Samples will be collected following strict handling and conservation protocols, ensuring the integrity and viability of the samples for future analyses. All samples will be coded to ensure participant confidentiality and privacy, and stored under controlled conditions. temperature and safety at the Laboratory of Neuroscience, Neuromodulation and Pain Study (LANNED). Participants will be informed about the retention and storage of samples during the informed consent process, ensuring that they are aware of and in agreement with the use prolonged storage of your biological samples for research purposes.

Execution Schedule

Stage Identification	Start (DD/MM/YYYY)	End Date (DD/MM/YYYY)
Data analysis	11/01/2024	04/30/2025
Article writing	04/30/2025	06/30/2025
Project submission	08/27/2024	08/27/2024
Project writing	06/28/2024	06/28/2024
Protocol execution	11/01/2024	11/15/2024
Training in the use of tools	10/01/2024	10/31/2024

Financial Budget

Budget Identification	Type	Value in Reais (R$)
Crushed ice	Costing	R$ 200.00
Elisa kit	Costing	R$ 1,500.00
Total in R$		R$ 1,700.00

Other information, justifications or considerations at the researcher's discretion:

Correction of Pending Issues: 1. Clarify regarding the collection of biological material (blood) - location of collection and disposal of biological materials. A: The following text was included in item 5.8.8: The collection of biological material, specifically blood samples, will be carried out in an appropriate environment within the training center, where all biosafety standards will be strictly followed. The procedure will be carried out by a qualified and experienced nurse, ensuring that the collection is done in a safe and hygienic manner. The collected material will be used exclusively for the analysis of creatine kinase (CK) levels and other possible biomarkers related to the study. After the collection, the blood will be stored in specific tubes and kept under controlled conditions until analysis. Biological materials and all items used during collection, such as needles, syringes, and other disposable equipment, will be properly discarded in containers suitable for biological waste, in accordance with current regulations on the disposal of hospital waste. 2. Enter all researchers in all documents (TCLE and detailed project). A: The inclusions were made. 3. Clarify in the TCLE that the participant, after randomization, you may be allocated to a group that will undergo cryotherapy or a group that will not undergo this intervention. In the TCLE presented apparently all volunteers will undergo cryotherapy. A: It was included in item 1 of the TCLE and in item 5.8.9 of the project detailed. 4. Clarify the need for two TCLEs for this research, considering that they were attached to the Plataforma Brasil two versions of the TCLE (TCLE_GELO.docx files), the first submitted on 06/28/24 and the other on 07/08/2024. A: A inclusion corrected on 07/08/2024, not excluding the version of 06/28/2024. However, in the present submission, these two

Machine Translated by Google
versions will be replaced by the corrected version after opinion.

Bibliography:

Alexander, J., Selfe, J., Greenhalgh, O., and Rhodes, D. (2021). Cryotherapy and compression in sports injury management: a scoping review. International Journal of Therapy and Rehabilitation 28, 1-19. Babraj, J. A., Vollaard, N. B., Keast, C., Guppy, F. M., Cottrell, G., and Timmons, J. A. (2009). Extremely short duration high intensity interval training substantially improves insulin action in young healthy males. BMC Endocr Disord 9, 3. Baker, L. B., Rollo, I., Stein, K. W., and Jeukendrup, A. E. (2015). Acute Effects of Carbohydrate Supplementation on Intermittent Sports Performance. Nutrients 7, 5733-5763. Bergeron, M.F., Nindl, B.C., Deuster, P.A., Baumgartner, N., Kane, S.F., Kraemer, W.J., Sexauer, L.R., Thompson, W.R., and O'connor, F.G. (2011). Consortium for Health and Military Performance and American College of Sports Medicine consensus paper on extreme conditioning programs in military personnel. Curr Sports Med Rep 10, 383-389. Borg, G. A. (1982). Psychophysical bases of perceived exertion. Med Sci Sports Exerc 14, 377-381. Bourdas, D.I., Souglis, A., Zacharakis, E.D., Geladas, N.D., and Travlos, A.K. (2021). Meta-Analysis of Carbohydrate Solution Intake during Prolonged Exercise in Adults: From the Last 45+ Years' Perspective. Nutrients 13. Broatch, J.R., Petersen, A., and Bishop, D.J. (2018). The Influence of Post-Exercise Cold-Water Immersion on Adaptive Responses to Exercise: A Review of the Literature. Sports Med 48, 1369- 1387. Bryer, SC, and Goldfarb, AH (2006). Effect of high dose vitamin C supplementation on muscle soreness, damage, function, and oxidative stress to eccentric exercise. Int J Sport Nutr Exerc Metab 16, 270-280. Cariappa, M. (2006). Designing Clinical Research: An Epidemiological Approach, 2nd edition, Stephen B Hulley (Ed.) et al.. Lippincott Williams & Wilkins, Philadelphia, USA (2001) http://www.amazon.com, 336 pages (softcover). Approx cost US$59.95. (available online at), ISBN: 0-7817-2218-7. Medical Journal Armed Forces India 62, 89. Carreker, J.D., and Grosicki, G.J. (2020). Physiological Predictors of Performance on the CrossFit "Murph" Challenge. Sports (Basel) 8. Cheung, K., Hume, P., and Maxwell, L. (2003). Delayed onset muscle soreness: treatment strategies and performance factors. Sports Med 33, 145-164. Connolly, D.A., Sayers, S.P., and Mchugh, M.P. (2003). Treatment and prevention of delayed onset muscle soreness. J Strength Cond Res 17, 197-208.

Desantana, J., Perissinotti, D., Oliveira Junior, J., Correia, L., Oliveira, C., and Fonseca, P. (2020). Definition of pain revised after four decades. Brazilian Journal Of Pain 3. Garber, CE, Blissmer, B., Deschenes, MR, Franklin, BA, Lamonte, MJ, Lee, IM, Nieman, DC, and Swain, DP (2011). American College of Sports Medicine position stand. Quantity and quality of exercise for developing and maintaining cardiorespiratory, musculoskeletal, and neuromotor fitness in apparently healthy adults: guidance for prescribing exercise. Med Sci Sports Exercise 43, 1334-1359. Gillen, J.B., and Gibala, M.J. (2014). Is high-intensity interval training a time-efficient exercise strategy to improve health and fitness? Appl Physiol Nutr Metab 39, 409-412. Gussoni, M., Moretti, S., Vezzoli, A., Genitoni, V., Giardini, G., Balestra, C., Bosco, G., Pratali, L., Spagnolo, E., Montorsi, M., and Mrakic-Sposta, S. (2023). Effects of Electrical Stimulation on Delayed Onset Muscle Soreness (DOMS): Evidences from Laboratory and In-Field Studies. J Funct Morphol Kinesiol 8. Hallal, P. C., Andersen, L. B., Bull, F. C., Guthold, R., Haskell, W., and Ekelund, U. (2012). Global physical activity levels: surveillance progress, pitfalls, and prospects. Lancet 380, 247-257. Hilbert, J.E., Sforzo, G.A., and Swensen, T. (2003). The effects of massage on delayed onset muscle soreness. Br J Sports Med 37, 72-75. Howatson, G., Van Someren, K., and Hortobágyi, T. (2007). Repeated bout effect after maximal eccentric exercise. Int J Sports Med 28, 557-563. Hunter, G. R., Seelhorst, D., and Snyder, S. (2003). Comparison of metabolism and heart rate responses to super slow vs. traditional resistance training. J Strength Cond Res 17, 76-81. Jackman, S.R., Witard, O.C., Jeukendrup, A.E., and Tipton, K.D. (2010). Branched-chain amino acid ingestion can improve soreness from eccentric exercise. Med Sci Sports Exerc 42, 962-970. Leeder, J., Gissane, C., Van Someren, K., Gregson, W., and Howatson, G. (2012). Cold water immersion and recovery from strenuous exercise: a meta-analysis. Br J Sports Med 46, 233-240. Lima, D., Sties, S., Gonzáles, A., Bündchen, D., Gomes Aquino, I., Carvalho, T., Neto, A., and Fontes, Y. (2016). QUESTIONNAIRE FOR EVALUATION OF MUSCULOSKELETAL PAIN IN EXERCISERS (Q-ADOM). Brazilian Journal of Sports Medicine 22, 374-380. Martinez, J., Grassi, D., and Marques, L. (2011). Analysis of the applicability of different pain questionnaires in three hospital settings: Outpatient clinic, ward and emergency unit. Brazilian journal of rheumatology 51, 299-303, 308. Mchugh, MP (2003). Recent advances in the understanding of the repeated bout effect: the protective effect against muscle damage from a single bout of eccentric exercise. Scand J Med Sci Sports 13, 88-97. Mccrae, G., Payne, A., Zelt, J., Scribbans, T., Jung, M., Little, J., and Gurd, B. (2012). Extremely low volume, whole-body aerobic-resistance training improves aerobic fitness and muscular endurance in females. Applied physiology, nutrition, and metabolism = Physiologie appliquee, nutrition et metabolism 37. Mizumura, K., and Taguchi, T. (2024). Neurochemical mechanism of muscular pain: Insight from the study on delayed onset muscle soreness. J Physiol Sci 74, 4. Molanorouzi, K., Khoo, S., and Morris, T. (2015). Motives for adult participation in physical activity: type of activity, age, and gender. BMC Public Health 15, 66. Nosaka, K., Clarkson, P. M., Mcguiggin, M. E., and Byrne, J. M. (1991). Time course of muscle adaptation after high force eccentric exercise. Eur J Appl Physiol Occup Physiol 63, 70-76. Pointon, M., Duffield, R., Cannon, J., and Marino, F. E. (2012). Cold water immersion recovery following intermittent-sprint exercise in the heat. Eur J Appl Physiol 112, 2483-2494. Shoepe, TC, Labrie, JW, Mello, GT, Leggett, AG, and Almstedt, HC (2020). Intensity of resistance training via self-reported history is critical in properly characterizing musculoskeletal health. BMC Musculoskelet Disord 21, 729. Smith, L. L. (1991). Acute inflammation: the underlying mechanism in delayed onset muscle soreness? Med Sci Sports Exerc 23, 542-551. Thompson, W. (2016). Worldwide survey of fitness trends for 2017. ACSM s Health & Fitness Journal 20, 8-17. Waller, M., Miller, J., and Hannon, J. (2011). Resistance Circuit Training: Its Application for the Adult Population. Strength & Conditioning Journal 33, 16-22. Wood, ER, Silva, AC, Baptista, GG, and Lüdorf, SMA (2022). Becoming a CrossFit practitioner: body management in and out of the box. Movement 28, e28038.

Upload Documents

File Attachments:

Type	File
Others	Questionnaire.docx
Others	Q_ADOM.pdf
Others	Questionnaire.docx
Others	Borg_Scale.docx
Others	Borg_Scale.docx

Others	TAI_CAPIXABA.pdf
Title Page	2024_facesheet_Marcelo_signed__2_.pdf
Detailed Project / Researcher Brochure	project_gaby_final.docx
Others	EVA.pdf
Detailed Project / Researcher Brochure	project_gaby_final.docx
Others	Questionnaire.docx

Machine Translated by Google

Receipt receipt	PB_COMPROVANTE_RECEPCAO_2374341.pdf
Others	EVA.pdf
Others	Brief_Pain_Inventory.pdf
Title Page	2024_facesheet_Marcelo_signed__2_.pdf
Detailed Project / Researcher Brochure	project_gaby_final.docx
TCLE / Terms of Assent / Justification of Absence	TCLE_ICE.docx
Detailed Project / Researcher Brochure	project_gaby_final.pdf
Others	EVA.pdf
Researchers' Statement	Declaration_Commitment.pdf
Others	Q_ADOM.pdf
TCLE / Terms of Assent / Justification of Absence	TCLE_ICE.docx
Basic Project Information	PB_BASIC_PROJECT_INFORMATION_2374341.pdf
TCLE / Terms of Assent / Justification of Absence	TCLE_ICE.docx
TCLE / Terms of Assent / Justification of Absence	TCLE_ICE.docx
Basic Project Information	PB_BASIC_PROJECT_INFORMATION_2374341.pdf
Detailed Project / Researcher Brochure	project_gaby_final.pdf
Others	TAI_CAPIXABA.pdf
TCLE / Terms of Assent / Justification of Absence	TCLE_ICE.docx
Detailed Project / Researcher Brochure	project_gaby_final.pdf
Others	Q_ADOM.pdf
Others	Brief_Pain_Inventory.pdf
Title Page	2024_facesheet_Marcelo_signed__2_.pdf
Others	TAI_CAPIXABA.pdf
Others	Borg_Scale.docx
Others	Brief_Pain_Inventory.pdf
TCLE / Terms of Assent / Justification of Absence	TCLE_ICE.docx
Researchers' Statement	Declaration_Commitment.pdf

Finish

Maintain confidentiality of the entire research project: Yes

Deadline: 1 year
