# Supplementary material for: Effectiveness of cold-water immersion vs. massage in reducing delayed-onset muscle soreness and enhancing recovery following CrossFit® Murph Workout: Randomized rial
Source: PLoS One. 2025 Aug 13;20(8):e0329892. doi: 10.1371/journal.pone.0329892 (PMC12349088; doi:10.1371/journal.pone.0329892)
Supplement: S3 File — Study protocol for the randomized controlled trial evaluating cryotherapy in delayed onset muscle soreness (DOMS) – Portuguese version. (PDF) [file pone.0329892.s003.pdf]

Projeto de Pesquisa:
AVALIAÇÃO DOS EFEITOS DA CRIOTERAPIA NA RECUPERAÇÃO DA DOR MUSCULAR TARDIA EM ATLETAS APÓS TREINO INTERVALADO DE ALTA INTENSIDADE NO WOD MURPH NO CROSSFIT: ENSAIO CLÍNICO, CONTROLADO E RANDOMIZADO

Informações Preliminares

Responsável Principal

CPF/Documento:	Nome: Marcelo Lourenço da Silva
Telefone: 1634215280	E-mail: marcelo.lourenco@unifal-mg.edu.br

Instituição Proponente

CNPJ: 17.879.859/0001-15	Nome da Instituição: UNIVERSIDADE FEDERAL DE ALFENAS - UNIFAL-MG

É um estudo internacional? Não

Equipe de Pesquisa

CPF/Documento	Nome
	Gabrielly Santos Pereira
	Josie Resende Torres da Silva

Área de Estudo

Grandes Áreas do Conhecimento

- Grande Área 4. Ciências da Saúde

Propósito Principal do Estudo

- Clínico

Título Público da Pesquisa: AVALIAÇÃO DOS EFEITOS DA CRIOTERAPIA NA RECUPERAÇÃO DA DOR MUSCULAR TARDIA EM ATLETAS APÓS TREINO INTERVALADO DE ALTA INTENSIDADE NO WOD MURPH NO CROSSFIT: ENSAIO CLÍNICO, CONTROLADO E RANDOMIZADO

Contato Público

CPF/Documento	Nome	Telefone	E-mail
[illegible]	Marcelo Lourenço da Silva	1634215280	marcelo.lourenco@unifal-mg.edu.br

Contato Científico: Marcelo Lourenço da Silva

Desenho de Estudo / Apoio Financeiro

Desenho do Estudo: Intervenção/Experimental

Condições de saúde ou problemas

Condição de saúde ou Problema
Dor muscular tardia

Descritores Gerais para as Condições de Saúde

CID1-10:Classificação Internacional de Doenças

Código CID	Descrição CID
M62	Outros transtornos musculares

Descritores Específicos para as Condições de

CID1-10:Classificação Internacional de Doenças

Código CID	Descrição CID
M62	Outros transtornos musculares

Tipo de Intervenção: Experimental

Natureza da Intervenção

- Outro — Crioterapia

Descritores da Intervenção

Descritores da Intervenção

Intervenções
Crioterapia

Fase

- Fase 1

Haverá uso de placebo ou a existência de grupos que não serão submetidos em nenhuma intervenção:

O uso de um placebo permite uma comparação mais precisa entre os efeitos da crioterapia real e as respostas psicossomáticas dos participantes, assegurando que quaisquer melhorias observadas no grupo de intervenção sejam realmente atribuídas à crioterapia e não ao efeito placebo.

Desenho:

Inicialmente, realizaremos uma coleta de dados basais de todos os atletas participantes para que possamos criar uma linha de referência. Logo, utilizaremos o questionário de avaliação da dor musculoesquelética (E-ADOM) para avaliar o nível de desconforto muscular dos participantes antes do início do treino. Em seguida, aplicamos o Inventário Breve de Dor (BPI), uma ferramenta que nos permite medir a intensidade da dor e seu impacto nas atividades diárias dos indivíduos. Complementarmente, a Escala Visual Analógica (EVA) será utilizada para que os participantes indiquem a intensidade da dor em uma linha de 0 a 10, proporcionando uma medida subjetiva da dor.
Para uma avaliação mais objetiva, utilizaremos a algometria, que medirá a sensibilidade à dor através da aplicação de pressão controlada sobre o músculo gastrocnêmio. Além disso, faremos uso de imagens termográficas para observar a temperatura do músculo gastrocnêmio, o que pode indicar inflamação ou outros processos fisiológicos. Por fim, coletaremos amostras de sangue para medir os níveis de creatina quinase (CK), uma enzima que serve como marcador de danos musculares. A análise desses níveis nos fornecerá dados importantes sobre o grau de lesão muscular causado pelo treino.
Após a coleta de dados basais, os atletas irão realizar o WOD Murph, que consiste em correr 1,6 quilômetros (1 milha), realizar 100 flexões na barra (pull-ups), 200 flexões de braço (push-ups), 300 agachamentos (air squats) e correr mais 1,6 quilômetros (1 milha), uma atividade física intensa que gera uma DMT.
Após a conclusão do treino, realizaremos uma intervenção com a crioterapia em imersão. Será disponibilizado uma banheira de gelo, na qual o atleta irá entrar cobrindo todo músculo gastrocnêmio. A aplicação de gelo nos músculos dos participantes visa reduzir a inflamação e a dor, promovendo uma recuperação mais rápida.
Para avaliar os efeitos da intervenção com crioterapia, repetiremos a coleta de todos os dados coletados pré treino em 24, 48 e 72 horas após o treino para possíveis conclusões.

Apoio Financeiro

CNPJ	Nome	E-mail	Telefone	Tipo
				Financiamento Próprio

Palavra Chave

Palavra-chave
treino intervalado de alta intensidade
dor muscular tardia
crioterapia

Detalhamento do Estudo

Resumo:

Atualmente, mais de 30% da população mundial é fisicamente inativa e um dos principais motivos é a limitação de tempo. Tal fato, traz implicações significativas para a saúde global, sugerindo recomendações de exercícios mais curtos e eficazes, como o treino intervalado de alta intensidade (HIIT). Esse tipo de treino tem a capacidade de melhorar a condição aeróbica, composição corporal e saúde cardiometabólica. Um exemplo de HIIT, é o treino "Murph" do CrossFit®, no qual combina modalidades aeróbicas e de resistência em circuitos intensos. O treino "Murph" do CrossFit, um dos mais desafiadores, frequentemente resulta em dor muscular tardia (DMT). Dessa forma, a imersão em gelo (crioterapia) se destaca como uma modalidade terapêutica viável para estudar, devido aos seus efeitos anti-inflamatórios e analgésicos promovendo uma recuperação rápida após o exercício. Portanto,o presente estudo tem como objetivo analisar o efeito da crioterapia como intervenção na recuperação da DMT após a prática o HIIT do workout of the day (WOD) Murph do CrossFit®. Esse estudo é um ensaio clínico controlado e randomizado, nos quais os participantes são divididos em grupos de controle (GC) e intervenção (GI). Os instrumentos de avaliação incluem a escala visual analógica de dor (EVA), escala de BORG, inventário breve de dor (BPI), questionário para avaliação da dor musculoesquelética de praticantes de exercício físico (Q-ADOM), algômetro, câmera termográfica e coleta de amostras de sangue para avaliar creatina quinase CK. Espera-se que os resultados contribuam para o desenvolvimento de protocolos mais precisos de intervenção com a crioterapia na DMT.

Introdução:

Mais de 30% da população mundial é fisicamente inativa, o que traz implicações significativas para a saúde global (Hallal et al., 2012). Atualmente, há um crescente reconhecimento de que as limitações de tempo enfrentadas por muitas pessoas ao iniciar programas de exercícios tornam inviáveis as recomendações anteriores, sugerindo que intervalos de tempo mais curtos podem ser mais apropriados (Molanorouzi et al., 2015). Além disso, exercícios de alta intensidade têm demonstrado proporcionar melhores resultados em termos de força e capacidade cardiorrespiratória (Garber et al., 2011).O treino intervalado de alta intensidade (HIIT) é geralmente definido como a repetição de séries curtas ou longas de exercícios intensos, intercaladas com períodos de recuperação ativa ou passiva. No entanto, não há consenso sobre a especificidade, frequência e duração do exercício necessárias para maximizar os benefícios para a saúde e prevenir doenças associadas ao sedentarismo (Gillen and Gibala, 2014). Este tipo de

treino pode melhorar a condição aeróbica, a composição corporal e a saúde cardiometabólica em diversas populações (Babraj et al., 2009).O treino HIIT é geralmente recomendado para ser realizado com exercícios de corpo inteiro e movimentos cíclicos, como caminhada, corrida, natação ou ciclismo, que não aumentam significativamente a força ou potência muscular (Waller et al., 2011). O treino multimodal combina várias modalidades, como exercícios de resistência aeróbica, força e circuitos. Há algumas evidências de que o treino contínuo de circuito pode induzir uma resposta cardiovascular, mas a intensidade usada geralmente varia de baixa a moderada (Hunter et al., 2003). Segundo McRae et al. (McRae et al., 2012), há evidências limitadas de que o HIIT de baixo volume pode ser tão eficaz quanto o treino aeróbico contínuo na melhoria da capacidade aeróbica e superior para ganhos de resistência muscular, embora este método ainda não tenha sido amplamente investigado.Um exemplo de HIIT é o CrossFit®, que é um treino multimodal de alta intensidade que incorpora padrões de movimento funcionais (multiarticulares) realizados em formato de circuito, com pausas curtas ou períodos de descanso entre exercícios ou grupos de exercícios (Bergeron et al., 2011) Desde 2013, o HIIT tem sido uma das três maiores tendências globais de fitness, com o CrossFit® sendo um dos principais fatores para essa popularidade, de acordo com a pesquisa anual da ACSM (Thompson, 2016). De acordo com Wood (Wood et al., 2022), o CrossFit® é eficaz em melhorar a aptidão cardiovascular e a composição corporal em praticantes de todos os níveis de condicionamento físico. No entanto, a metodologia do CrossFit® também enfrenta críticas por não seguir rigorosamente os princípios de treinamento estabelecidos, e há pouca pesquisa sobre as respostas específicas aos diferentes tipos de treino dentro dessa metodologia.A Associação Internacional para o Estudo da Dor (IASP) define a dor como uma experiência sensorial e emocional desagradável associada a, ou semelhante àquela associada a, lesão real ou potencial dos tecidos (DeSantana et al., 2020). Nesse contexto, a dor muscular tardia (DMT) consiste na sensação de dor ou desconforto nos músculos que normalmente, aumenta de intensidade nas primeiras 24 horas, e atinge o pico entre 24 e 48 após o exercício (Cheung et al., 2003). Esse tipo de dor é um sinal de que os músculos estão se adequando em resposta a atividades físicas intensas, principalmente as que envolvem contrações excêntricas, como a fase de descida do agachamento, levantamento de peso na musculação ou a corrida em declive. Durante essas atividades, os níveis de creatina quinase (CK) no sangue aumentam, indicando dano muscular.Um exemplo notável de treino que pode induzir DMT é o "Murph", um dos treinos mais emblemáticos do CrossFit, conhecido por sua exigência física e mental. Criado em memória do Tenente Michael P. Murphy, um militar da Marinha dos Estados Unidos morto em combate, este Workout of the Day (WOD) consiste em correr 1,6 quilômetros (1 milha), realizar 100 flexões na barra (pull-ups), 200 flexões de braço (push-ups), 300 agachamentos (air squats) e correr mais 1,6 quilômetros (1 milha). O objetivo é completar o treino no menor tempo possível, sendo um exercício de alta intensidade aeróbica, mas influenciado pelo desempenho anaeróbio e pela capacidade de recuperação de exercícios de alta intensidade (Carreker and Grosicki, 2020). Devido à sua intensidade e ao grande volume de exercícios, o Murph pode induzir a DMT, muitas vezes dificultando a realização de treinos subsequentes devido ao desconforto.Analisar a ocorrência de DMT e seus efeitos após o WOD Murph, uma atividade de alta intensidade, é essencial. A recuperação (conhecido como recovery) para DMT, logo após o treinamento como uma medida de intervenção, deve ser considerado aprimorando assim, estratégias para recuperação dos atletas. Nosso projeto visa entender de que forma a imersão em gelo aplicada logo após a prática dessa atividade intensa podem minimizar a DMT. A imersão em gelo (crioterapia) se destaca como uma modalidade terapêutica viável para estudar. Vários estudos indicam que a crioterapia pode reduzir a inflamação e a dor muscular tardia, promovendo uma recuperação mais rápida após HIIT. Por exemplo, um estudo realizado por Leeder et al. (Leeder et al., 2012) demonstrou que a imersão em água fria pode reduzir a dor muscular de início tardio e acelerar a recuperação muscular . Além disso, a crioterapia é amplamente utilizada e aceita em ambientes esportivos, e há um corpo significativo de pesquisas que respaldam sua eficácia (Alexander et al., 2021) . Portanto, investigar a imersão em gelo pode proporcionar dados relevantes e aplicáveis para melhorar a recuperação dos atletas após atividades de alta intensidade como o WOD Murph.Dessa forma, este projeto científico visa preencher lacunas no conhecimento sobre a DMT após a realização do HIIT no WOD Murph no CrossFit, verificando ainda se com a intervenção a dor persiste. Espera-se que os resultados destas investigações contribuam para o desenvolvimento de protocolos terapêuticos mais eficazes e melhorias significativas na qualidade de treino dos atletas afetados por essa condição dolorosa.

Hipótese:

A hipótese do estudo é que a aplicação de crioterapia em imersão após a realização do treino intenso "Murph" do CrossFit® reduzirá significativamente a dor muscular tardia (DMT) e a inflamação, promovendo uma recuperação mais rápida e eficiente nos atletas, em comparação com aqueles que não recebem a intervenção com crioterapia.

Objetivo Primário:

Investigar os efeitos da crioterapia como intervenção de recuperação na manifestação da DMT em atletas após a realização de atividades físicas intensas no CrossFit.

Objetivo Secundário:

Verificar a intensidade da dor percebida na DMT pós treino intenso, Murph, utilizando o algômetro como método de investigação. Verificar se a DMT altera a percepção analgésica na EVA e BPI após o treino Murph.Verificar se a DMT altera a percepção de dor musculoesquelética com o questionário Q-ADOM após o treino Murph.Avaliar os níveis da proteína quinase CK como biomarcador da DMT após o treino Murph.Verificar por meio da termografia se há alteração na temperatura dos músculos antes e após treinamento.

Metodologia Proposta:

Metodologia Proposta:Delineamento do Estudo: Este estudo será um ensaio clínico, controlado e randomizado, projetado para avaliar a eficácia da crioterapia na recuperação da dor muscular tardia (DMT) em atletas de CrossFit após a realização do treino Murph.Local e População de Estudo: O estudo será conduzido no centro de treinamento Capixaba, com apoio do Laboratório de Neurociência, Neuromodulação e Estudo da Dor (LANNED), localizado em Alfenas, Minas Gerais. A população do estudo será composta por 30 atletas que participam do treinamento no box Capixaba.Amostra: Serão selecionados 30 atletas que participaram do treinamento Murph no box Capixaba. Os participantes serão recrutados através de convites feitos diretamente no box Capixaba, onde os atletas costumam treinar regularmente. Serão realizadas apresentações breves antes ou após as sessões de treino para explicar os objetivos e a importância do estudo, bem como os critérios de inclusão e exclusão. Além disso, serão distribuídos panfletos informativos e afixados cartazes nas dependências do box, contendo detalhes sobre o estudo e informações de contato dos pesquisadores. Os indivíduos serão divididos em 2 grupos: o grupo controle e o grupo intervenção, alocados através de um processo de randomização realizado utilizando o site Research Randomizer.Randomização das Amostras: Os voluntários serão divididos em GC e GI através de randomização, utilizando o site Research Randomizer para garantir a alocação aleatória dos participantes.Cálculo Amostral: O cálculo amostral será realizado de acordo com Cariappa (2006), estimando 15 voluntários por grupo para garantir um poder estatístico de 85% (ß=0,20) e nível de significância de $p<0,05$.Instrumentos de Avaliação: Diversos instrumentos serão utilizados para avaliar a dor e a recuperação dos participantes:1. Questionário de Avaliação para Aplicação do Protocolo: Coleta informações sobre suplementação, alimentação, frequência de treinos, uso de modalidades terapêuticas e qualidade do sono.2. Escala Visual Analógica (EVA): Avalia a intensidade da dor, graduada de 0 a 10.3. Escala de BORG: Avalia a percepção de esforço físico, utilizando uma escala de 0 a 10.4. Inventário Breve de Dor (BPI): Avalia a intensidade da dor e seu impacto nas atividades diárias.5. Questionário para Avaliação da Dor Musculoesquelética em Praticantes de Exercício (Q-ADOM): Identifica as características da dor e sua influência sobre a prática de exercício físico.6. Algômetro: Mede a sensibilidade à dor aplicando pressão controlada.7.

Câmera Termográfica: Detecta a radiação infravermelha emitida pelo corpo, visualizando a temperatura da superfície da pele.8. Quantificação da Proteína CK: Medição dos níveis de creatina quinase (CK) no sangue como marcador de danos musculares.Intervenção: A intervenção consistirá em sessões de imersão em água fria (crioterapia) imediatamente após o treino Murph. A temperatura da água será mantida abaixo de 15°C e a duração da imersão será de 10 minutos. A sessão de crioterapia envolverá a imersão das pernas dos atletas em um recipiente adequado, garantindo que todas as áreas musculares principais envolvidas no exercício sejam tratadas.Procedimentos Experimentais: Inicialmente, será realizada uma coleta de dados basais utilizando o E-ADOM, BPI, EVA, algometria, termografia e amostras de sangue para medir CK. Após a coleta de dados basais, os atletas realizarão o WOD Murph. Após o treino, será realizada a intervenção com crioterapia. Os dados serão coletados novamente 24, 48 e 72 horas após o treino para avaliar os efeitos da intervenção na DMT.

Critério de Inclusão:

Os participantes devem ter entre 18 e 45 anos de idade e pelo menos 6 meses de experiência regular em CrossFit, treinando no mínimo três vezes por semana. É essencial que os atletas sejam considerados saudáveis e não apresentem lesões musculoesqueléticas atuais ou crônicas que possam interferir na realização do WOD Murph ou na aplicação da crioterapia. Além disso, os participantes não devem ter realizado o WOD "Murph" ou exercícios de intensidade similar nas quatro semanas anteriores ao início do estudo.
Todos os participantes devem fornecer consentimento informado por escrito, indicando que compreendem os procedimentos, riscos e benefícios do estudo. Também é necessário que estejam disponíveis para as sessões de treinamento, aplicação da crioterapia e avaliações subsequentes dentro do período estabelecido pelo estudo. O índice de massa corporal (IMC) dos atletas deve estar entre 18,5 e 30 kg/m², para excluir extremos de massa corporal que poderiam influenciar os resultados. Além disso, os participantes não devem ter usado crioterapia regularmente como método de recuperação nos três meses anteriores ao estudo.
É importante que os atletas demonstrem a capacidade de seguir as instruções e os protocolos do estudo de forma consistente e correta. Por fim, os participantes devem ser capazes de ler e compreender o idioma no qual o estudo e o consentimento informado são apresentados, garantindo que entendam todas as instruções e requisitos do estudo.

Critério de Exclusão:

Participantes serão excluídos se apresentarem qualquer condição médica que possa ser exacerbada pelo exercício intenso ou pela exposição ao frio, como doenças cardiovasculares, respiratórias ou metabólicas graves. Atletas com histórico de lesões musculoesqueléticas crônicas ou recentes que possam interferir na realização do WOD Murph ou na aplicação da crioterapia também serão excluídos.
Além disso, serão excluídos os indivíduos que tenham realizado o WOD Murph ou exercícios de intensidade similar nas quatro semanas anteriores ao início do estudo, para evitar efeitos de treinamentos anteriores. Participantes que usaram crioterapia regularmente como método de recuperação nos três meses anteriores ao estudo também não serão incluídos, para garantir que a intervenção seja uma novidade para todos os sujeitos e seus efeitos possam ser avaliados de forma mais clara.
Atletas que não possam fornecer consentimento informado por escrito, ou que não compreendam completamente os procedimentos, riscos e benefícios do estudo, serão excluídos. Da mesma forma, indivíduos que não estejam disponíveis para participar de todas as sessões de treinamento, aplicação da crioterapia e avaliações subsequentes dentro do período estabelecido pelo estudo serão desconsiderados. Aqueles que apresentem um índice de massa corporal (IMC) fora do intervalo de 18,5 a 30 kg/m², para excluir extremos de massa corporal que poderiam influenciar os resultados, também serão excluídos.
Participantes que não demonstrem a capacidade de seguir as instruções e os protocolos do estudo de forma consistente e correta serão excluídos, para assegurar a uniformidade na aplicação dos métodos de recuperação. Indivíduos que não possam ler e compreender o idioma no qual o estudo e o consentimento informado são apresentados serão excluídos, garantindo que todos os participantes entendam completamente as instruções e os requisitos do estudo. Além disso, serão excluídos os atletas que estejam utilizando suplementação alimentar específica para recuperação muscular ou anti-inflamatórios, pois essas substâncias podem interferir nos resultados da pesquisa.

Riscos:

A crioterapia, embora amplamente utilizada e geralmente segura, pode apresentar alguns riscos potenciais. A exposição a temperaturas extremamente baixas pode causar desconforto temporário, incluindo sensação de frio intenso, formigamento ou dormência na pele. Em casos raros, a imersão em água fria pode resultar em lesões cutâneas como queimaduras por frio ou congelamento se a temperatura não for monitorada adequadamente. Indivíduos com condições médicas preexistentes, como problemas cardiovasculares, respiratórios ou metabólicos, podem estar em maior risco de complicações adversas devido à vasoconstrição e à resposta sistêmica ao frio. Portanto, a avaliação médica prévia será realizada para identificar e excluir esses indivíduos.
Os participantes também podem experimentar um aumento temporário na rigidez muscular ou desconforto após a imersão em água fria, especialmente se não estiverem acostumados com esse tipo de terapia. Além disso, a coleta de sangue para medir os níveis de creatina quinase (CK) pode causar desconforto leve, hematomas ou infecção no local da punção venosa, embora esses riscos sejam mínimos quando procedimentos adequados de assepsia são seguidos.

Benefícios:

Os benefícios da participação no estudo incluem a possibilidade de redução da DMT e melhoria na recuperação muscular, permitindo que os atletas retornem às suas atividades físicas com maior rapidez e menos desconforto. Os participantes terão acesso a uma intervenção terapêutica potencialmente eficaz que pode não estar disponível para eles fora do contexto do estudo. Além disso, os dados obtidos podem proporcionar informações valiosas sobre a eficácia da crioterapia, contribuindo para o desenvolvimento de práticas de recuperação mais eficazes no campo do CrossFit e do esporte em geral.
Os participantes também podem se beneficiar de um monitoramento mais detalhado de sua condição física e de sua resposta ao exercício, recebendo feedback e orientação de profissionais qualificados. Este acompanhamento pode ajudá-los a entender melhor como seu corpo responde ao treino intenso e às estratégias de recuperação, potencialmente melhorando sua prática de exercícios e prevenindo lesões futuras.
Em termos de contribuição científica, os resultados do estudo podem beneficiar a comunidade esportiva mais ampla, fornecendo evidências que podem orientar a implementação de estratégias de recuperação eficazes para outros atletas.
Como medidas minimizadoras dos riscos, primeiramente, todos os participantes passarão por uma avaliação médica prévia para identificar possíveis contra indicações para a crioterapia. Essa avaliação incluirá um exame físico detalhado e uma revisão do histórico médico para excluir indivíduos com condições cardiovasculares, respiratórias ou metabólicas que possam ser exacerbadas pela exposição ao frio.
Durante as sessões de crioterapia, a temperatura da água será rigorosamente monitorada e mantida abaixo de 15°C, utilizando termômetros calibrados para garantir precisão. A imersão será limitada a um máximo de 10 minutos para evitar qualquer dano à pele ou tecidos subjacentes.
Além disso, a supervisão por profissionais qualificados durante toda a intervenção garantirá que qualquer desconforto ou efeito adverso seja

imediatamente identificado e tratado. Os participantes serão instruídos a relatar quaisquer sensações anormais ou desconfortos durante a imersão, permitindo a interrupção imediata da sessão se necessário.
Para minimizar os riscos associados à coleta de sangue para a medição dos níveis de creatina quinase (CK), serão seguidos procedimentos rigorosos de assepsia. A coleta será realizada por profissionais treinados utilizando materiais esterilizados para reduzir o risco de infecção. Além disso, os locais de punção serão cuidadosamente selecionados e monitorados para prevenir hematomas e outras complicações menores.
Os participantes receberão instruções detalhadas sobre os procedimentos do estudo e as medidas de segurança antes do início da intervenção. Essas instruções incluem orientações sobre a importância da hidratação, aquecimento e resfriamento adequados antes e após os treinos e a sessão de crioterapia. Também serão fornecidas informações sobre como monitorar e relatar quaisquer efeitos adversos após as sessões.
Além das medidas imediatas de segurança, será estabelecido um sistema de monitoramento contínuo para acompanhar os participantes ao longo do estudo. Isso incluirá avaliações regulares da dor muscular e outros sintomas, utilizando métodos padronizados como a Escala Visual Analógica (EVA), o Inventário Breve de Dor (BPI) e o Questionário para Avaliação da Dor Musculoesquelética em Praticantes de Exercício (Q-ADOM). O uso de algômetros e câmeras termográficas proporcionará avaliações objetivas da dor e da inflamação, garantindo que qualquer alteração significativa na condição dos participantes seja detectada precocemente.
No caso de qualquer evento adverso ou complicação, os participantes terão acesso imediato a cuidados médicos apropriados. Protocolos de emergência serão estabelecidos e os profissionais envolvidos no estudo estarão preparados para lidar com qualquer situação inesperada.

Metodologia de Análise de Dados:

Inicialmente, serão realizadas análises descritivas para sumarizar as características demográficas e clínicas dos participantes, bem como os resultados das medidas de dor. Para comparar os níveis de dor antes e após a intervenção com crioterapia, serão utilizados testes T pareados ou análises de variância (ANOVA) de medidas repetidas, dependendo da normalidade dos dados. Se os dados não seguirem uma distribuição normal, testes não paramétricos como o teste de Wilcoxon serão aplicados.
Além disso, análises de regressão múltipla poderão ser realizadas para identificar possíveis fatores que influenciam a eficácia da crioterapia, como idade, sexo, experiência em CrossFit e nível de intensidade do treino. Análises de correlação serão conduzidas para investigar as relações entre diferentes métodos de avaliação da dor e os níveis de CK. A significância estatística será estabelecida em um nível de $p < 0,05$. Todos os dados serão analisados utilizando o software estatístico SPSS 20.

Desfecho Primário:

Espera-se que esta pesquisa venha contribuir para protocolos e métodos de avaliação mais precisos sobre a DMT. Fornecimento de dados concretos para treinadores, fisioterapeutas, e atletas sobre como otimizar a recuperação pós-exercício, melhorando a performance e a aderência aos programas de treinamento. Além de preencher uma lacuna significativa na literatura quanto à eficácia das estratégias de recuperação pósexercício.

Tamanho da Amostra no 30
Data do Primeiro Recrutamento: 08/11/2024

Países de Recrutamento

País de Origem do Estudo	País	Nº de participantes da pesquisa
Sim	BRASIL	30

Outras Informações

Haverá uso de fontes secundárias de dados (prontuários, dados demográficos, etc)?

Não

Informe o número de indivíduos abordados pessoalmente, recrutados, ou que sofrerão algum tipo de intervenção neste centro de pesquisa:

30

Grupos em que serão divididos os participantes da pesquisa neste centro

ID Grupo	Nº de Indivíduos	Intervenções a serem realizadas
Controle	15	Não farão crioterapia
Crioterapia	15	Crioterapia

O Estudo é Multicêntrico no Brasil?

Não

Propõe dispensa do TCLE?

Não

Haverá retenção de amostras para armazenamento em banco?

Sim

Justificativa:

Sim, haverá retenção de amostras para armazenamento em banco. As amostras de sangue coletadas para medir os níveis de creatina quinase (CK) serão armazenadas em um banco de amostras biológicas. Esse armazenamento permitirá futuras análises adicionais que possam surgir como relevantes para o estudo ou para pesquisas subsequentes relacionadas à dor muscular tardia (DMT) e recuperação muscular.As amostras serão coletadas seguindo protocolos rigorosos de manuseio e conservação, assegurando a integridade e viabilidade das amostras para futuras análises. Todas as amostras serão codificadas para garantir a confidencialidade e privacidade dos participantes, e armazenadas em condições controladas de temperatura e segurança no Laboratório de Neurociência, Neuromodulação e Estudo da Dor (LANNED).Os participantes serão informados sobre a retenção e armazenamento das amostras durante o processo de consentimento informado, garantindo que estejam cientes e de acordo com o uso prolongado das suas amostras biológicas para fins de pesquisa.

Cronograma de Execução

Identificação da Etapa	Início (DD/MM/AAAA)	Término (DD/MM/AAAA)
Análise dos dados	01/11/2024	30/04/2025
Escrita do artigo	30/04/2025	30/06/2025
Submissão do projeto	27/08/2024	27/08/2024
Escrita do projeto	28/06/2024	28/06/2024
Execução do protocolo	01/11/2024	15/11/2024
Treinamento da utilização das ferramentas	01/10/2024	31/10/2024

Orçamento Financeiro

Identificação de Orçamento	Tipo	Valor em Reais (R$)
Gelo picado	Custeio	R$ 200,00
Kit elisa	Custeio	R$ 1.500,00
Total em R$		R$ 1.700,00

Outras informações, justificativas ou considerações a critério do pesquisador:

Correção das Pendências: 1. Esclarecer a respeito de coleta de material biológico (sangue) - local da coleta e descarte dos materiais biológicos. R: Foi realizada a inclusão do seguinte texto no item 5.8.8: A coleta de material biológico, especificamente de amostras de sangue, será realizada em um ambiente apropriado dentro do centro de treinamento, onde todas as normas de biossegurança serão rigorosamente seguidas. O procedimento será realizado por um enfermeiro qualificado e experiente, garantindo que a coleta seja feita de forma segura e higiênica. O material coletado será utilizado exclusivamente para a análise dos níveis de creatina quinase (CK) e outras possíveis biomarcadores relacionados ao estudo. Após a coleta, o sangue será armazenado em tubos específicos e mantido em condições controladas até a análise. Os materiais biológicos e todos os itens utilizados durante a coleta, como agulhas, seringas, e outros equipamentos descartáveis, serão devidamente descartados em recipientes apropriados para resíduos biológicos, de acordo com as regulamentações vigentes de descarte de resíduos hospitalares. 2. Inserir todos os pesquisadores em todos os documentos (TCLE e projeto detalhado). R: Foram realizadas as inclusões. 3. Esclarecer no TCLE que o participante, após randomização, poderá ser alocado em grupo que será submetido a crioterapia ou em grupo que não será submetido a esta intervenção. No TCLE apresentado aparentemente todos os voluntários serão submetidos à crioterapia. R: Foi incluído no item 1 do TCLE e no item 5.8.9 do projeto detalhado. 4. Esclarecer quanto à necessidade de dois TCLEs para a presente pesquisa, considerando-se que foram anexados na Plataforma Brasil duas versões do TCLE (arquivos TCLE_GELO.docx), sendo o primeiro submetido em 28/06/24 e o outro em 08/07/2024. R: Foi realizada uma inxlusão corrigida em 08/07/2024, não sendo excluída a versão de 28/06/2024. Entretanto, na presente submissão, essas duas versões serão substituídas pela versão corrigida após parecer.

Bibliografia:

Alexander, J., Selfe, J., Greenhalgh, O., and Rhodes, D. (2021). Cryotherapy and compression in sports injury management: a scoping review. International Journal of Therapy and Rehabilitation 28, 1-19. Babraj, J.A., Vollaard, N.B., Keast, C., Guppy, F.M., Cottrell, G., and Timmons, J.A. (2009). Extremely short duration high intensity interval training substantially improves insulin action in young healthy males. BMC Endocr Disord 9, 3. Baker, L.B., Rollo, I., Stein, K.W., and Jeukendrup, A.E. (2015). Acute Effects of Carbohydrate Supplementation on Intermittent Sports Performance. Nutrients 7, 5733-5763. Bergeron, M.F., Nindl, B.C., Deuster, P.A., Baumgartner, N., Kane, S.F., Kraemer, W.J., Sexauer, L.R., Thompson, W.R., and O'connor, F.G. (2011). Consortium for Health and Military Performance and American College of Sports Medicine consensus paper on extreme conditioning programs in military personnel. Curr Sports Med Rep 10, 383-389. Borg, G.A. (1982). Psychophysical bases of perceived exertion. Med Sci Sports Exerc 14, 377-381. Bourdas, D.I., Souglis, A., Zacharakis, E.D., Geladas, N.D., and Travlos, A.K. (2021). Meta-Analysis of Carbohydrate Solution Intake during Prolonged Exercise in Adults: From the Last 45+ Years' Perspective. Nutrients 13. Broatch, J.R., Petersen, A., and Bishop, D.J. (2018). The Influence of Post-Exercise Cold-Water Immersion on Adaptive Responses to Exercise: A Review of the Literature. Sports Med 48, 13691387. Bryer, S.C., and Goldfarb, A.H. (2006). Effect of high dose vitamin C supplementation on muscle soreness, damage, function, and oxidative stress to eccentric exercise. Int J Sport Nutr Exerc Metab 16, 270-280. Cariappa, M. (2006). Designing Clinical Research: An Epidemiological Approach, 2nd edition, Stephen B Hulley (Ed.) et al.. Lippincott Williams & Wilkins, Philadelphia, USA (2001) http://www.amazon.com, 336 pages
(softcover). Approx cost US$59.95. (available online at), ISBN: 0-7817-2218-7. Medical Journal Armed Forces India 62, 89. Carreker, J.D., and Grosicki, G.J. (2020). Physiological Predictors of Performance on the CrossFit "Murph" Challenge. Sports (Basel) 8. Cheung, K., Hume, P., and Maxwell, L. (2003). Delayed onset muscle soreness : treatment strategies and performance factors. Sports Med 33, 145-164. Connolly, D.A., Sayers, S.P., and Mchugh, M.P. (2003). Treatment and prevention of delayed onset muscle soreness. J Strength Cond Res 17, 197-208. Desantana, J., Perissinotti, D., Oliveira Junior, J., Correia, L., Oliveira, C., and Fonseca, P. (2020). Definition of pain revised after four decades. Brazilian Journal Of Pain 3. Garber, C.E., Blissmer, B., Deschenes, M.R., Franklin, B.A., Lamonte, M.J., Lee, I.M., Nieman, D.C., and Swain, D.P. (2011). American College of Sports Medicine position stand. Quantity and quality of exercise for developing and maintaining cardiorespiratory, musculoskeletal, and neuromotor fitness in apparently healthy adults: guidance for prescribing exercise. Med Sci Sports Exerc 43, 1334-1359. Gillen, J.B., and Gibala, M.J. (2014). Is high-intensity interval training a time-efficient exercise strategy to improve health and fitness? Appl Physiol Nutr Metab 39, 409-412. Gussoni, M., Moretti, S., Vezzoli, A., Genitoni, V., Giardini, G., Balestra, C., Bosco, G., Pratali, L., Spagnolo, E., Montorsi, M., and Mrakic-Sposta, S. (2023). Effects of Electrical Stimulation on Delayed Onset Muscle Soreness (DOMS): Evidences from Laboratory and In-Field Studies. J Funct Morphol Kinesiol 8. Hallal, P.C., Andersen, L.B., Bull, F.C., Guthold, R., Haskell, W., and Ekelund, U. (2012). Global physical activity levels: surveillance progress, pitfalls, and prospects. Lancet 380, 247-257. Hilbert, J.E., Sforzo, G.A., and Swensen, T. (2003). The effects of massage on delayed onset muscle soreness. Br J Sports Med 37, 72-75. Howatson, G., Van Someren, K., and Hortobágyi, T. (2007). Repeated bout effect after maximal eccentric exercise. Int J Sports Med 28, 557-563. Hunter, G.R., Seelhorst, D., and Snyder, S. (2003). Comparison of metabolic and heart rate responses to super slow vs. traditional resistance training. J Strength Cond Res 17, 76-81. Jackman, S.R., Witard, O.C., Jeukendrup, A.E., and

Tipton, K.D. (2010). Branched-chain amino acid ingestion can ameliorate soreness from eccentric exercise. Med Sci Sports Exerc 42, 962-970. Leeder, J., Gissane, C., Van Someren, K., Gregson, W., and Howatson, G. (2012). Cold water immersion and recovery from strenuous exercise: a meta-analysis. Br J Sports Med 46, 233-240. Lima, D., Sties, S., Gonzáles, A., Bündchen, D., Gomes Aquino, I., Carvalho, T., Neto, A., and Fontes, Y. (2016). QUESTIONÁRIO PARA AVALIAÇÃO DA DOR MUSCULOESQUELÉTICA EM PRATICANTES DE EXERCÍCIO (Q-ADOM). Revista Brasileira de Medicina do Esporte 22, 374-380. Martinez, J., Grassi, D., and Marques, L. (2011). Analysis of the applicability of different pain questionnaires in three hospital settings: Outpatient clinic, ward and emergency unit. Revista brasileira de reumatologia 51, 299-303, 308. Mchugh, M.P. (2003). Recent advances in the understanding of the repeated bout effect: the protective effect against muscle damage from a single bout of eccentric exercise. Scand J Med Sci Sports 13, 88-97. Mcrae, G., Payne, A., Zelt, J., Scribbans, T., Jung, M., Little, J., and Gurd, B. (2012). Extremely low volume, whole-body aerobic-resistance training improves aerobic fitness and muscular endurance in females. Applied physiology, nutrition, and metabolism = Physiologie appliquee, nutrition et metabolisme 37. Mizumura, K., and Taguchi, T. (2024). Neurochemical mechanism of muscular pain: Insight from the study on delayed onset muscle soreness. J Physiol Sci 74, 4. Molanorouzi, K., Khoo, S., and Morris, T. (2015). Motives for adult participation in physical activity: type of activity, age, and gender. BMC Public Health 15, 66. Nosaka, K., Clarkson, P.M., Mcguiggin, M.E., and Byrne, J.M. (1991). Time course of muscle adaptation after high force eccentric exercise. Eur J Appl Physiol Occup Physiol 63, 70-76. Pointon, M., Duffield, R., Cannon, J., and Marino, F.E. (2012). Cold water immersion recovery following intermittent-sprint exercise in the heat. Eur J Appl Physiol 112, 2483-2494. Shoepe, T.C., Labrie, J.W., Mello, G.T., Leggett, A.G., and Almstedt, H.C. (2020). Intensity of resistance training via self-reported history is critical in properly characterizing musculoskeletal health. BMC Musculoskelet Disord 21, 729. Smith, L.L. (1991). Acute inflammation: the underlying mechanism in delayed onset muscle soreness? Med Sci Sports Exerc 23, 542-551. Thompson, W. (2016). Worldwide survey of fitness trends for 2017. ACSM s Health & Fitness Journal 20, 8-17. Waller, M., Miller, J., and Hannon, J. (2011). Resistance Circuit Training: Its Application for the Adult Population. Strength & Conditioning Journal 33, 16-22. Wood, E.R., Silva, A.C., Baptista, G.G., and Lüdorf, S.M.A. (2022). Tornando-se um praticante de CrossFit: gerenciamentos do corpo dentro e fora dos boxes. Movimento 28, e28038.

Upload de Documentos

Arquivo Anexos:

Tipo	Arquivo
Outros	Questionario.docx
Outros	Q_ADOM.pdf
Outros	Questionario.docx
Outros	Escala_de_Borg.docx
Outros	Escala_de_Borg.docx
Outros	TAI_CAPIXABA.pdf
Folha de Rosto	2024_folhaDeRosto_Marcelo_assinado__2_.pdf
Projeto Detalhado / Brochura Investigador	projeto_gaby_final.docx
Outros	EVA.pdf
Projeto Detalhado / Brochura Investigador	projeto_gaby_final.docx
Outros	Questionario.docx
Comprovante de Recepção	PB_COMPROVANTE_RECEPCAO_2374341.pdf
Outros	EVA.pdf
Outros	Inventario_Breve_de_Dor.pdf
Folha de Rosto	2024_folhaDeRosto_Marcelo_assinado__2_.pdf
Projeto Detalhado / Brochura Investigador	projeto_gaby_final.docx
TCLE / Termos de Assentimento / Justificativa de Ausência	TCLE_GELO.docx
Projeto Detalhado / Brochura Investigador	projeto_gaby_final.pdf
Outros	EVA.pdf
Declaração de Pesquisadores	Declaracao_Compromisso.pdf
Outros	Q_ADOM.pdf
TCLE / Termos de Assentimento / Justificativa de Ausência	TCLE_GELO.docx
Informações Básicas do Projeto	PB_INFORMAÇÕES_BÁSICAS_DO_PROJETO_2374341.pdf
TCLE / Termos de Assentimento / Justificativa de Ausência	TCLE_GELO.docx

TCLE / Termos de Assentimento / Justificativa de Ausência	TCLE_GELO.docx
Informações Básicas do Projeto	PB_INFORMAÇÕES_BÁSICAS_DO_PROJETO_2374341.pdf
Projeto Detalhado / Brochura Investigador	projeto_gaby_final.pdf
Outros	TAI_CAPIXABA.pdf
TCLE / Termos de Assentimento / Justificativa de Ausência	TCLE_GELO.docx
Projeto Detalhado / Brochura Investigador	projeto_gaby_final.pdf
Outros	Q_ADOM.pdf
Outros	Inventario_Breve_de_Dor.pdf
Folha de Rosto	2024_folhaDeRosto_Marcelo_assinado__2_.pdf
Outros	TAI_CAPIXABA.pdf
Outros	Escala_de_Borg.docx
Outros	Inventario_Breve_de_Dor.pdf
TCLE / Termos de Assentimento / Justificativa de Ausência	TCLE_GELO.docx
Declaração de Pesquisadores	Declaracao_Compromisso.pdf

Finalizar

Manter sigilo da integra do projeto de pesquisa: Sim

Prazo: 1 ano
